# Analyses on clustering of the conserved residues at protein-RNA interfaces and its application in binding site identification

**DOI:** 10.1186/s12859-020-3398-9

**Published:** 2020-02-17

**Authors:** Zhen Yang, Xueqing Deng, Yang Liu, Weikang Gong, Chunhua Li

**Affiliations:** College of Life Science and Bioengineering, Beijing University of Technology, Beijing, 100124 China

**Keywords:** Protein-RNA interfaces, Conserved residues, Clustering characteristics, Hot spot residues, Binding site identification

## Abstract

**Background:**

The maintenance of protein structural stability requires the cooperativity among spatially neighboring residues. Previous studies have shown that conserved residues tend to occur clustered together within enzyme active sites and protein-protein/DNA interfaces. It is possible that conserved residues form one or more local clusters in protein tertiary structures as it can facilitate the formation of functional motifs. In this work, we systematically investigate the spatial distributions of conserved residues as well as hot spot ones within protein-RNA interfaces.

**Results:**

The analysis of 191 polypeptide chains from 160 complexes shows the polypeptides interacting with tRNAs evolve relatively rapidly. A statistical analysis of residues in different regions shows that the interface residues are often more conserved, while the most conserved ones are those occurring at protein interiors which maintain the stability of folded polypeptide chains. Additionally, we found that 77.8% of the interfaces have the conserved residues clustered within the entire interface regions. Appling the clustering characteristics to the identification of the real interface, there are 31.1% of cases where the real interfaces are ranked in top 10% of 1000 randomly generated surface patches. In the conserved clusters, the preferred residues are the hydrophobic (Leu, Ile, Met), aromatic (Tyr, Phe, Trp) and interestingly only one positively charged Arg residues. For the hot spot residues, 51.5% of them are situated in the conserved residue clusters, and they are largely consistent with the preferred residue types in the conserved clusters.

**Conclusions:**

The protein-RNA interface residues are often more conserved than non-interface surface ones. The conserved interface residues occur more spatially clustered relative to the entire interface residues. The high consistence of hot spot residue types and the preferred residue types in the conserved clusters has important implications for the experimental alanine scanning mutagenesis study. This work deepens the understanding of the residual organization at protein-RNA interface and is of potential applications in the identification of binding site and hot spot residues.

## Introduction

Protein-RNA interactions play important roles in a wide variety of cellular processes, such as regulation of gene expression, RNA splicing, protein synthesis and virus assembly [1, 2]. Proteins are under certain evolutionary pressures for selecting their RNA partners in a crowded cellular environment [3]. Consequently, the interaction interfaces experience relatively higher evolutionary pressures, and therefore interface residues are generally more conserved [4, 5].

How are these conserved residues organized at protein-RNA interfaces? Are they scattered across the interface, or clustered together in three dimensions? How are the preferences of residue types and hot spot residues (contributing significantly to the binding free energy) for different interface regions? Whether can these characteristics be used to identify the real interface? These questions are not quite clear currently.

The previous study has shown that for protein-protein interactions, 96.7 and 86.7% of the interfaces of homodimers and heterocomplexes respectively have the conserved residues clustered within the overall interface regions [6]. And Ahmad et al. found that for the proteins interacting with DNAs, about half of the observed conserved residue clusters are in the interfaces with DNAs and the remaining are in the interfaces with proteins or ligands, or embedded in the structural scaffolds [7].

The higher packing density of the conserved residues within the interface may suggest the cooperativity between them in the cluster. It is likely that the most stabilizing residues or putative hot spot residues are those that occur as clusters of conserved residues, contributing more to the stability and function of interactions than others, which has been confirmed in protein-DNA interfaces by Ahmad et al. [7]. Landgraf et al. and Madabushi’s group have found that in protein tertiary structures and enzyme active sites, the evolutionary conserved residues also occur clustered together [8, 9]. These conserved residues form one or more localized clusters within the tertiary structure or interface, which will facilitate the formation of “functional motifs”. Additionally, the relationship between hot spot residues and conserved residue clusters is a significant topic in the study of protein structure and stability [10].

In this work, we investigate the spatial distribution characteristics and amino acid composition of the evolutionary conserved residues within protein-RNA interfaces, and also explore the relationship between interface hot spots and the conserved residue clusters. The results show that the conserved residues are not randomly distributed within the interface, but are obviously clustered together, which can be used to identify the real protein-RNA interfaces. Furthermore, the identification of these clusters will be a useful guide for mutagenesis studies to determine the appropriate hot spot regions.

## Materials and methods

### Construction of dataset of protein-RNA interfaces

A total of 1031 protein-RNA complexes were extracted from the Protein Data Bank (PDB), which were solved by X-ray diffraction with resolution better than 3.0 Å [11] (June 2018). After excluding the complexes that have protein chains of less than 30 amino acids or RNA chains of less than 5 nucleotides, we clustered the redundant complexes that contain proteins with > 30% sequence identity and the same RNAs. From each cluster, the structure with the highest resolution was chosen as the representative. The cases that are composed of redundant proteins and different RNA molecules were kept for considering different interfaces. Thus we obtained 182 non-redundant protein-RNA complexes.

As the sequence entropy is needed in this work, the complexes where the protein has enough homologous sequences to calculate the sequence conservation will be remained. Multiple sequence alignments (MSA) were carried out by ClustalW [12, 13] against the UniRef90 database [14] with default parameters and Gonnet substitution matrix [15] for all protein chains in 182 complexes. ClustalW, developed by Thompson, improves the sensitivity of progressive multiple sequence alignments through sequence weighting, position-specific gap penalties and weight matrix choice [12, 13]. As one of the most widely and classically used MSA programs [13], ClustalW has been used in many studies such as protein phylogenetic and conserved motif analyses [16], evolutionary distance analyses [17], and residue level molecular function prediction [18].

For the aligned sequences, we removed the sequences with sequence identity less than 45% or missing residues more than five. A protein that has more than five homologous sequences retained was put into the dataset. Eventually, we constructed a dataset of 160 protein-RNA complexes (183 interfaces) involving 191 polypeptide chains. According to the molecular functions, the dataset is divided into five different classes: protein-mRNA (8), protein-tRNA (53), protein-rRNA (23), protein-viral RNA (7) and protein-other RNA (69) complexes (Table S1 in supplementary materials).

### Determination of protein interface, non-interface surface and interior residues

The non-interface surface residues are defined as those having relative solvent accessible surface area (SASA, calculated with NACCESS [19]) > 5% in the complex structure. A residue’s relative SASA is computed as a ratio of its SASA in the complex to its SASA in the extended tripeptide (Ala-*X*-Ala), where *X* is the concerned residue. The interface residues of proteins are those that lose more than 0.1 Å^2^ of SASA upon complexation with RNA. And those residues that are not the interface and the surface ones are protein interior residues [20].

### Calculation of sequence conservation

The protein sequence conservations at each interior, interface and non-interface surface residue position are calculated as the Shannon entropy (*s*) in a set of homologous protein sequences [21]:

1 $$ s(i)=-\sum {p}_i(k)\cdot lo{g}_2\left({p}_i(k)\right), $$

where *p*_*i*_(*k*) is the probability that a residue of type *k* occurs at the *i*th position in the sequence alignment. The lower value in sequence entropy of a position hints that it has suffered a higher evolutionary pressure.

Here, the amino acids are grouped into seven classes based on the similarity of their environment in protein structures, and mutations within a given class are assumed to be conservative and do not cause a penalty [22]. The following is the classification of amino acid classes: (1) Thr, Gly, Ser; (2) Val, Ala, Ile, Cys, Met, Leu; (3) Gln, Asn; (4) Glu, Asp; (5) Trp, Tyr, Phe, Pro; (6) His; and (7) Lys, Arg [23]. The sequence entropy s(i) ranges between 0 (there is only one class of residues occurring at position i) to ~ 2.81 (there are seven classes of residues that are equally distributed at position i in the sequence aligment). For each protein chain in complex, <s > is the mean value of sequence entropy over all the residue positions.

### Identification of conserved interface residues

For each interface (owning *n* residues), an average value of sequence entropy is computed:

2 $$ <s{>}_{int}=\left(\sum s(i)\right)/n, $$

Three criteria are utilized to define the conserved interface residues, which have different stringent levels. Here the purpose of using different criteria is to see that with the decrease of the number of interface conserved residues, what changes occur to the clustering property of conserved residues within the interface? The conserved interface residue is defined as that with sequence entropy value (1) lower than the average value (<*s* > _*int*_) of the interface where it occurs, (2) lower than half of the average value (<*s* > _*int*_/2), and (3) equal to 0.0, namely, the fully conserved residues, respectively.

### Measure of the spatial clustering degree

We use the average inverse distance among all pairs of residues in a set of residues to evaluate the spatial clustering degree of that set [24]:

3 $$ {M}_s=<1/r>=\frac{1}{N_{pairs}}\sum \limits_{i=1}^{N_s-1}\sum \limits_{j=i+1}^{N_s}\left(1/{r}_{ij}\right), $$

where *r*_*ij*_ is the distance between Ca atoms of residues *i* and *j*, *N*_*pairs*_ is the number of different residue pairs, and *N*_*s*_ is the number of residues in the set. The larger the value of *M*_*s*_, the greater the spatial clustering degree of the residues in the set. The *M*_*s*_ value for the whole set can not be influenced obviously by one or a few outlier positions, which is the benefit of the inverse-distance based formula.

For each interface, we define a ratio *ρ* to reflect the clustering degree of the conserved interface residues relative to all interface residues:

4 $$ \rho ={M}_{s, cons}/{M}_{s,\mathit{\operatorname{int}}}, $$

where *M*_*s,cons*_ and *M*_*s,int*_ are the spatial clustering degrees of the subsets of conserved and entire interface residues, respectively. *ρ* can be used to evaluate whether or not (and to what extent) the evolutionary conserved residues are clustered within the interface. The conserved interface residues are clustered, then *ρ* > 1.0. Here, we remove the interfaces that own an isolated conserved residue when measuring the size of the conserved residue cluster.

### Identification of sub-clusters of conserved interface residues

We found the conserved residues are spatially clustered together, rather than scattered in the structure. And within the entire interface, the conserved residues may constitute be consist of one or more sub-clusters. The average linkage method [25] is used to identify the number of sub-clusters. We adopt the threshold distance 20 Å involved in the algorithm which equals to half the mean value of the maximum distances between any two conserved residue atoms in all the interfaces.

### Generation of surface patches and comparison of the clustering of conserved residues at the interface with that at surface patches

We utilize three methods to generate surface patches. From method 1 to 3, the generated surface patch in a protein is more and more close to its own interface in size. Method 1: for all the proteins with their partner RNAs removed, NACCESS is conducted on their atomic coordinates and surface residues are identified based on the same definition mentioned above. In generating a surface patch process, we take a random surface residue (represented by its Ca atom) and then choose all the surface residues that are less than a fixed radius away from the taken residue as belonging to the surface patch with the taken residue as the center. Between any two atoms of all the interfaces, the mean maximum distance is 40 Å, and thus we use 20 Å- half of 40 Å to produce surface patches. Method 2: for each protein we use its own cutoff, rather than a uniform one, according to its interface size. Method 3: surface neighbors meeting two criteria the distance cutoff, and a vector constraint are selected around the randomly selected central residue [26]. The vector constraint avoids generating the surface patches which include the residues from “opposite sides” of a protein. In this step, we compute a ‘solvent’ vector (pointing into the solvent) for each surface residue of a protein. The direction of the ‘solvent’ vector of a surface residue is from the geometrical center of its nearest ten residue neighbors to its Ca atom. We remove the residue out from the patch if the angle between the solvent vectors of it and the central residue of the patch is ≥110° during generating a surface patch.

Each of the three procedures thus defines a number of contiguous, overlapping patches of surface residues, roughly similar in size to the interface region. For the generated surface patches from each procedure, conserved residues within each patch are selected and the *M*_*s*_ values (Eq. 3) for both the conserved and the overall residues in the patch are calculated. The calculation is repeated for each patch. Finally, for each of the three procedures, all the surface patches from a protein are ranked in descending order of *ρ* (Eq. 4) and the rank of the real interface in relation to all the other surface patches is found out.

### Experimental alanine scanning mutagenesis data

A set of 41 protein-RNA complexes with experimental alanine scanning mutagenesis data on the interface residues are available in the dbAMEPNI database [27]. Hot spot residues are selected from the 139 interface residues based on three criteria respectively, i.e. experimental ΔΔG value ≥1.0, ≥ 1.5, and ≥ 2.0 kcal/mol (Table S2 in supplementary materials).

## Results

### Evolution of polypeptide chains in protein-RNA complexes

**Fig. 1:**
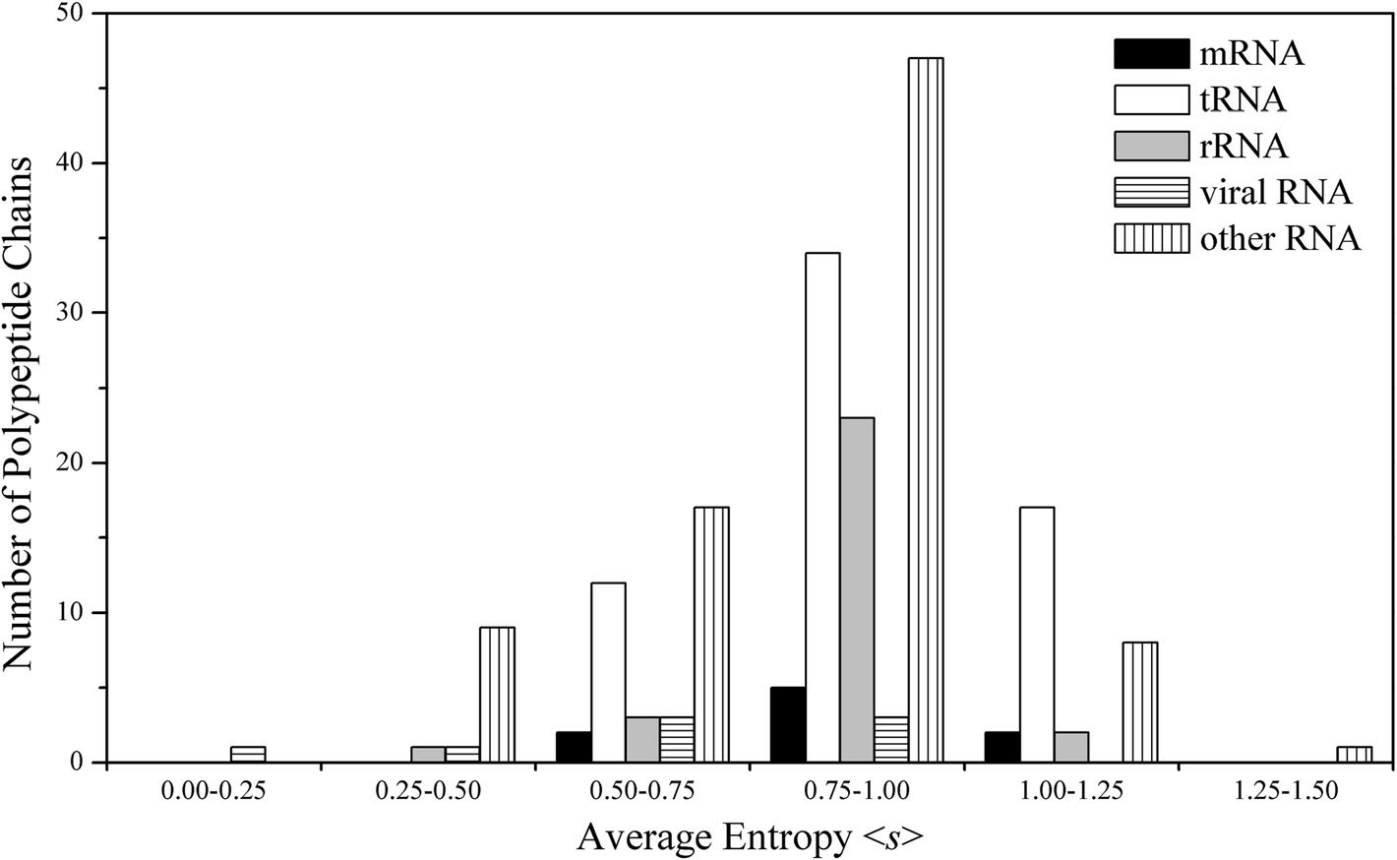
Distributions of the mean sequence entropy <*s* > of polypeptide chains in different function classes of protein-RNA complexes

To explore the difference in evolutionary conservation of polypeptide chains with difference functions, we calculated the average sequence entropy of each chain (see the section of Calculation of sequence conservation in Materials and Methods) and its distribution for the five function classes of protein-RNA complexes, as shown in Fig. 1 and Table S1 (detailed values) in supplementary materials, respectively. The average sequence entropy <*s* > varies from 0.23 (4WSB_A, bat influenza polymerase) to 1.36 (5I9F_A, designed pentatricopeptide repeat protein) in the entire dataset, which suggests that some RNA binding proteins (RBP) evolve rapidly compared to others. From the average values of <*s* > in different types of complexes, the protein chains binding with tRNAs have a largest average value (0.90) compared with other types of protein chains, indicating that the polypeptides interacting with tRNAs evolve relatively rapid, which is consistent with previous work [20]. Additionally, for the RBP with multiple chains, the evolutions of different chains also present evident difference. For the complex of human m1A58 methyltransferase with tRNA (PDB code: 5CCB), we can see that its chain A has a lower evolutionary pressure (<*s* > = 1.00), while chain B, participating in the main interaction with tRNA, experiences a higher evolutionary pressure (<*s* > = 0.68).

### Evolutions of interiors, interfaces and non-interface surfaces in protein-RNA complexes

**Fig. 2:**
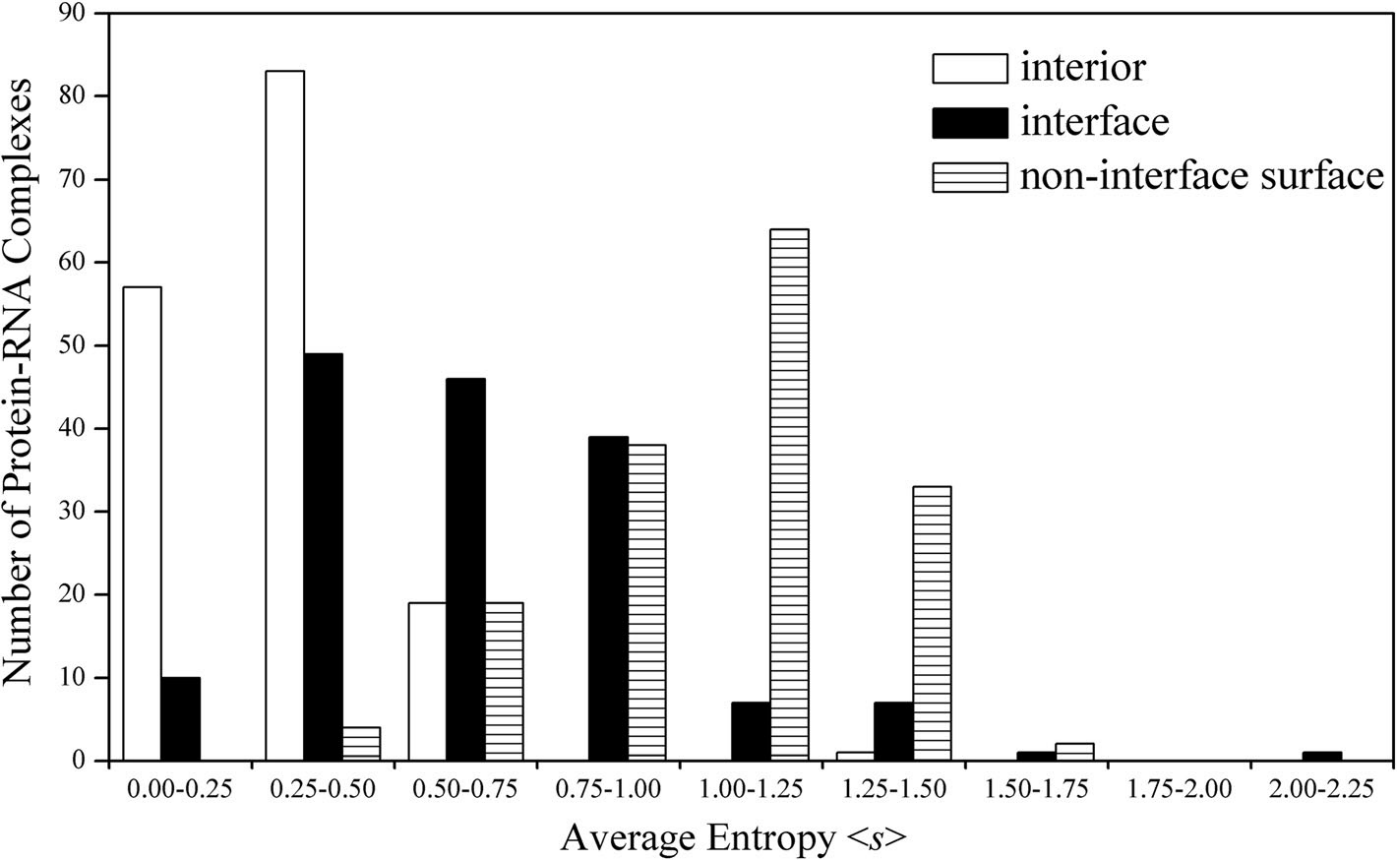
Distributions of mean sequence entropy of the residues in interior, interface and non-interface surface regions in protein-RNA complexes

We concern whether different parts of proteins experience different sequence evolutionary pressures. In order to detect this point, we calculated the average sequence entropies of the interior, interface and non-interface surface residues for different types of protein-RNA complexes. The results are shown in Table 1 and Fig. 2. From Table 1, the three types of residues occupy 25.10, 11.61 and 63.29% of all residues respectively, which are approximately equal to the corresponding values in different classes of protein-RNA complexes. From Table 1, the average entropies <*s* > of the residues presented in protein interiors and solvent exposed surfaces are equal to 0.34 (the lowest) and 1.05 (the highest) respectively, and the value in between - 0.64 corresponds to the residues at protein-RNA interfaces. The distribution data in Fig. 2 also show that the residues at interfaces are more conserved than those at solvent exposed surfaces, and protein interior residues are the most conserved, which is originally similar in all classes of complexes (see Table 1).

**Table 1:** Occurring percentage and average sequence entropy of interior, interface and non-interface surface residues

Parameters	Protein peptides complexed with					All peptides
	mRNA	tRNA	rRNA	viral RNA	other RNA	
% residues in						
interior	21.50	26.72	24.35	21.60	24.36	25.10
interface	14.76	11.45	13.27	10.80	11.24	11.61
non-interface surface	63.76	61.83	62.38	67.60	64.40	63.29
Average < *s*>						
interior	0.18	0.40	0.34	0.19	0.29	0.34
interface	0.50	0.73	0.67	0.48	0.57	0.64
non-interface surface	0.92	1.16	1.05	0.62	1.02	1.05

Additionally, it should be pointed out that the residues in the three regions of the protein chains interacting with tRNAs possess significantly higher entropies than those in corresponding regions of the other four types of complexes (Table 1), which is consistent with the above result that the polypeptides interacting with tRNAs evolve relatively rapidly. Taking five cases belonging to the five different classes of protein-RNA complexes for example, Figure S1 in supplementary materials illustrates the conservation distributions of the residues at protein surfaces. In all the five structures, the protein surface that interacts with RNA is relatively prone to owning higher conservative property than the non-interface surface.

### Relative conservations of amino acid residues in interiors, interfaces and non-interface surfaces

**Fig. 3:**
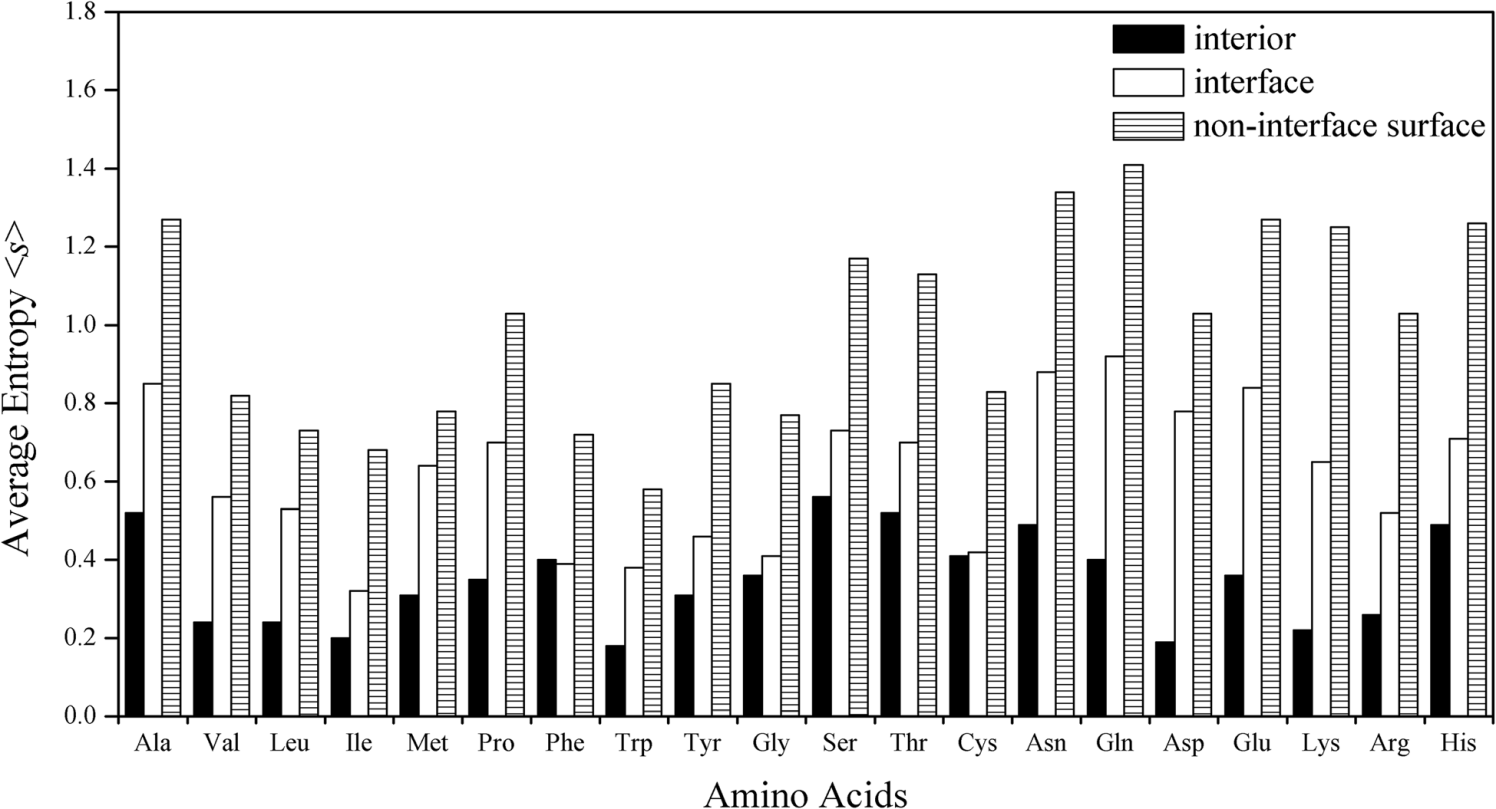
Mean entropy of different types of amino acid residues in protein interior, interface and non-interface surface regions

Next we want to know the relative conservations of 20 types of amino acid residues at protein interiors, interfaces and solvent exposed surfaces. We calculated the average sequence entropies of 20 types of amino acid residues in the three regions for all complexes, and the results are shown in Fig. 3 and Table S3 in supplementary materials (detailed values). From Fig. 3, regarding to the speed of evolution, compared with all types of residues at interfaces and interiors, the corresponding ones at non-interface surfaces evolve faster, and still all types at interiors evolve slower than the corresponding ones at interfaces except for Phe. At interfaces, remarkably, the aromatic residues Trp, Phe, and Tyr, as well as Ile, Gly, Cys and Arg (owning the smallest values of <s>: 0.38, 0.39, 0.46, 0.32, 0.41, 0.42 and 0.52, relatively) are more conserved than other types of amino acid residues. At non-interface surfaces, the two neutral polar amino acid residues Asn and Gln (having the highest values of <*s*>: 1.34 and 1.41, relatively) are more frequently mutated compared with others. As for interiors, the hydrophobic amino acids Trp, Val, Leu and Ile (0.18, 0.24, 0.24 and 0.20) and charged Asp, Lys and Arg (0.19, 0.22 and 0.26) are most conserved. Hydrophobic amino acids are prone to occurring inside proteins [28] while charged amino acids existing in the interiors may serve a functional role.

### Clustering of conserved residues in protein-RNA interfaces

**Fig. 4:**
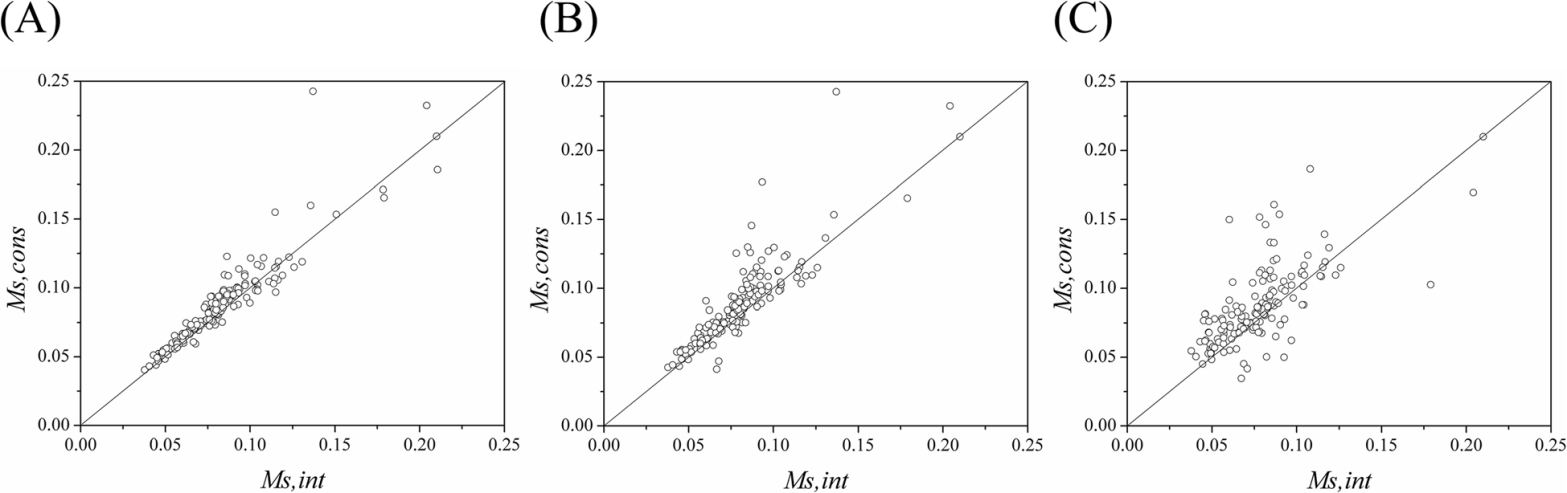
Plots of *M*_*s,cons*_ versus *M*_*s,int*_ for all protein-RNA complexes according to the three conserved residue definitions of gradually more stringent levels: the conserved residue with sequence entropy lower than the average value (<*s* > _*int*_) of the interface where it occurs (**a**), lower than half of the average value (<*s* > _*int*_/2) (**b**), and equal to 0.0, namely, the fully conserved residue (**c**), respectively

The next question we concern is that the evolutionary conserved residues in protein-RNA interfaces are scattered or gathered together in three-dimensional structures. We calculated the spatial clustering degree *M*_*s*_ (Eq. 3) for both subsets of the conserved (*M*_*s,cons*_) and all the interface residues (*M*_*s,int*_), and their ratio *ρ* (Eq. 4). Fig. 4 and Table 2 show the corresponding results obtained based on different criteria of conserved residues (see Table S4 for detailed values in supplementary materials).

**Table 2:** Parameters describing the clustering of conserved interface residues

Criteria of conserved residues	Average^a^			Num of interfaces^b^	
	*M*_*s,int*_	*M*_*s,cons*_	*ρ*	Total	with *M*_*s,cons*_ > *M*_*s,int*_
*s* < <*s* > _*int*_	0.082 (0.03)	0.087 (0.03)	1.06 (0.10)	180	140
*s* < (<*s* > _*int*_/2)	0.080 (0.03)	0.088 (0.03)	1.11 (0.18)	172	135
*s* = 0.0	0.078 (0.03)	0.087 (0.03)	1.15 (0.28)	150	114

^a^ Standard deviations are in parentheses

^b^ A smaller number of interfaces is obtained when using more stringent definitions of conserved residues

**Fig. 5:**
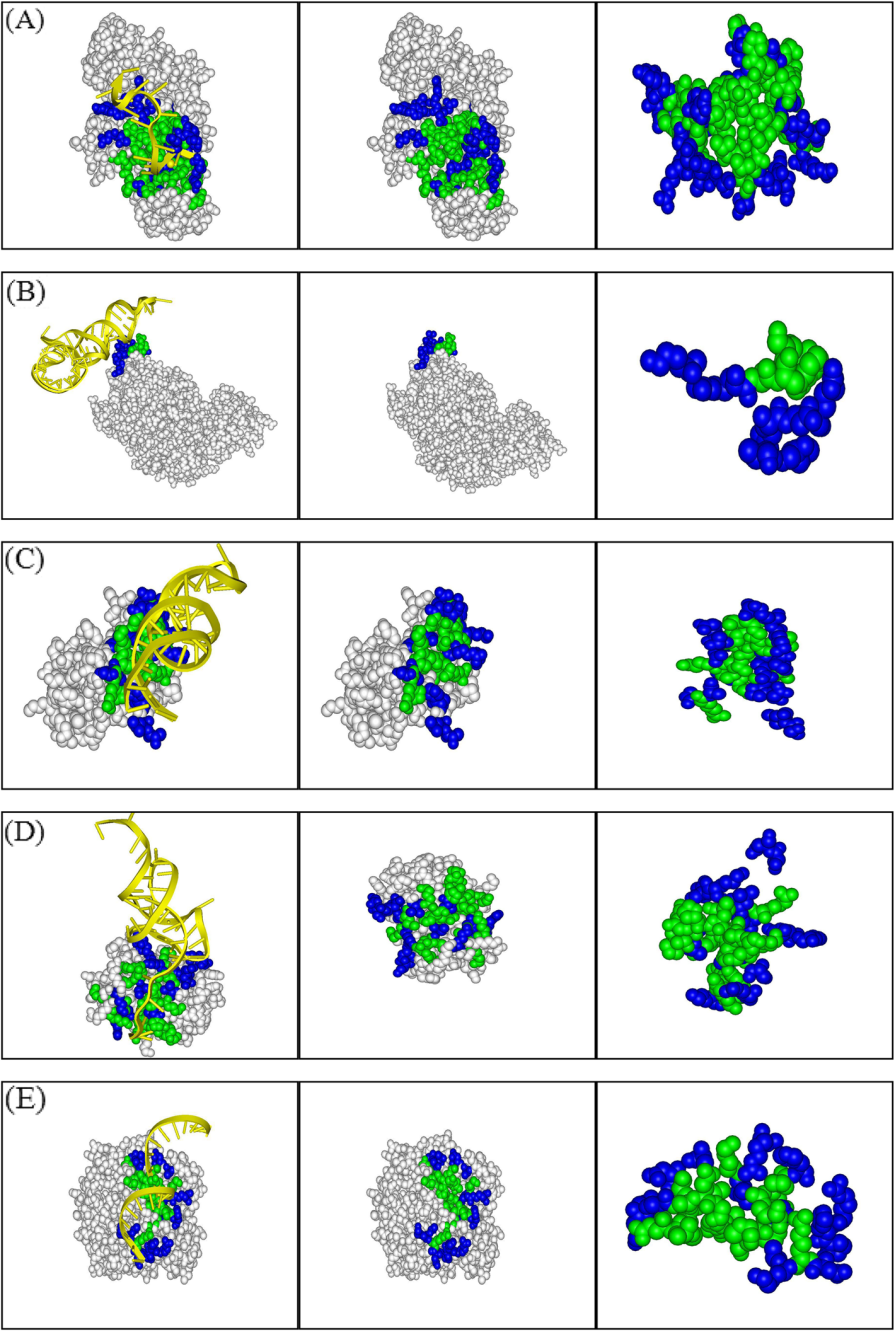
Representative examples of protein interfaces showing the clustering characteristics of evolutionary conserved residues. Protein is shown in CPK (grey), the conserved residues are in green and non-conserved ones in blue. (**a**) The SXL-UNR translation regulatory complex (PDB code: 4qqb). In the first panel, RNA is in yellow and in the other two, it is removed to clearly show the clustering property of the conserved residues. (**b**) The prolyl-tRNA synthetase from thermus thermophilus complexed with tRNA (PDB code: 1h4q). (**c**) The ribosomal protein s8-rRNA complex (PDB code: 1i6u). (**d**) The bacterial protein-RNA toxin-antitoxin system (PDB code: 4rmo). (**e**) The human adenosine bound to dsRNA (PDB code: 5ed2)

Fig. 4 displays *M*_*s,cons*_ and *M*_*s,int*_ for each interface. From Fig. 4, most of the points are lying above the diagonal, resulting in the mean value of *ρ* 1.06, 1.11 and 1.15 corresponding to the gradually more stringent criteria of conserved residues (*s* < <*s* > _*int*_, *s* < (<*s* > _*int*_/2) and *s* = 0.0), respectively, which indicates that for most of the interfaces *M*_*s,cons*_ is greater than *M*_*s,int*_ with *p*-value 0.0311, 0.0044 and 0.0427. For all the protein-RNA complexes, there are 77.8% (140/180), 78.5% (135/172) and 76.0% (114/150) of interfaces where a *ρ* value of greater than 1.0 is obtained (see Table 2). From the analyses above, the results imply that the conserved interface residues are more spatially clustered relative to the entire interface residues, and this tendency holds true for the definitions of conserved residues with different stringent levels. Additionally, for different types of protein-RNA complexes, the tendency also holds true (Table S5 in supplementary materials). A few representative examples of the interfaces where the conserved residues are clearly clustered together are shown in Fig. 5.

### Formation of multiple conserved residue sub-clusters in larger interfaces

The maintenance of the stability of biological systems requires synergy among different functional units. Then we concern whether multiple conserved residue sub-clusters form at an interface. We calculated the number of sub-clusters composed of the conserved interface residues for each interface with the average linkage method. The distribution of the numbers of conserved residue sub-clusters in interfaces as a function of the interface area is displayed in Figure S2 in supplementary materials. From Figure S2, it can be observed that almost the interfaces (153/180) owning single cluster have areas less than 3000 Å^2^, and all (7) except for two which possess three or more sub-clusters have the areas > 3000 Å^2^. Thus, most of the protein-RNA interfaces own one single cluster of conserved residues, and roughly, the larger interfaces often form multiple detached sub-clusters. Three representative cases whose interfaces own respectively one, two and three sub-clusters of conserved residues are shown in Figure S3 in supplementary materials. For the larger interfaces, it may be important to form distinct binding sub-clusters (or “hot regions”) that interact cooperatively via hydrogen bonds and salt bridges for protein-RNA interaction stability.

### Sub-cluster size

The conserved residues can occur alone, or organize into multiple sub-clusters containing different numbers of conserved residues. We analyzed the distribution of the sub-cluster sizes (i.e., the number of conserved residues) for the 204 different sub-clusters in all interfaces, and the result is shown in Figure S4 in supplementary materials. On average, a sub-cluster consists of 10 conserved residues. There are only 1.5% (3/204) of sub-clusters composing of a single isolated conserved residue. Therefore, it is evident that most of conserved residues prefer to be clustered together rather than to occur isolated.

### Preferred amino acid types in conserved residue clusters

Certain types of amino acid residues may have propensities to occur in the conserved residue clusters. Here, the propensity is evaluated using the relative enrichment *E*_*x*_ which defines a probability of type *X* of the 20 amino acid residue types occurring in the conserved interface subsets compared to the whole interfaces.

$$ {E}_X=\frac{\frac{\mathrm{No}.\mathrm{of}\ X\ \mathrm{in}\ \mathrm{conserved}\ \mathrm{subset}}{\mathrm{Total}\ \mathrm{no}.\mathrm{of}\ \mathrm{conserved}\ \mathrm{residues}}}{\frac{\mathrm{No}.\mathrm{of}\ X\ \mathrm{in}\ \mathrm{interface}}{\mathrm{Total}\ \mathrm{no}.\mathrm{of}\ \mathrm{interface}\ \mathrm{residues}}} $$

**Fig. 6:**
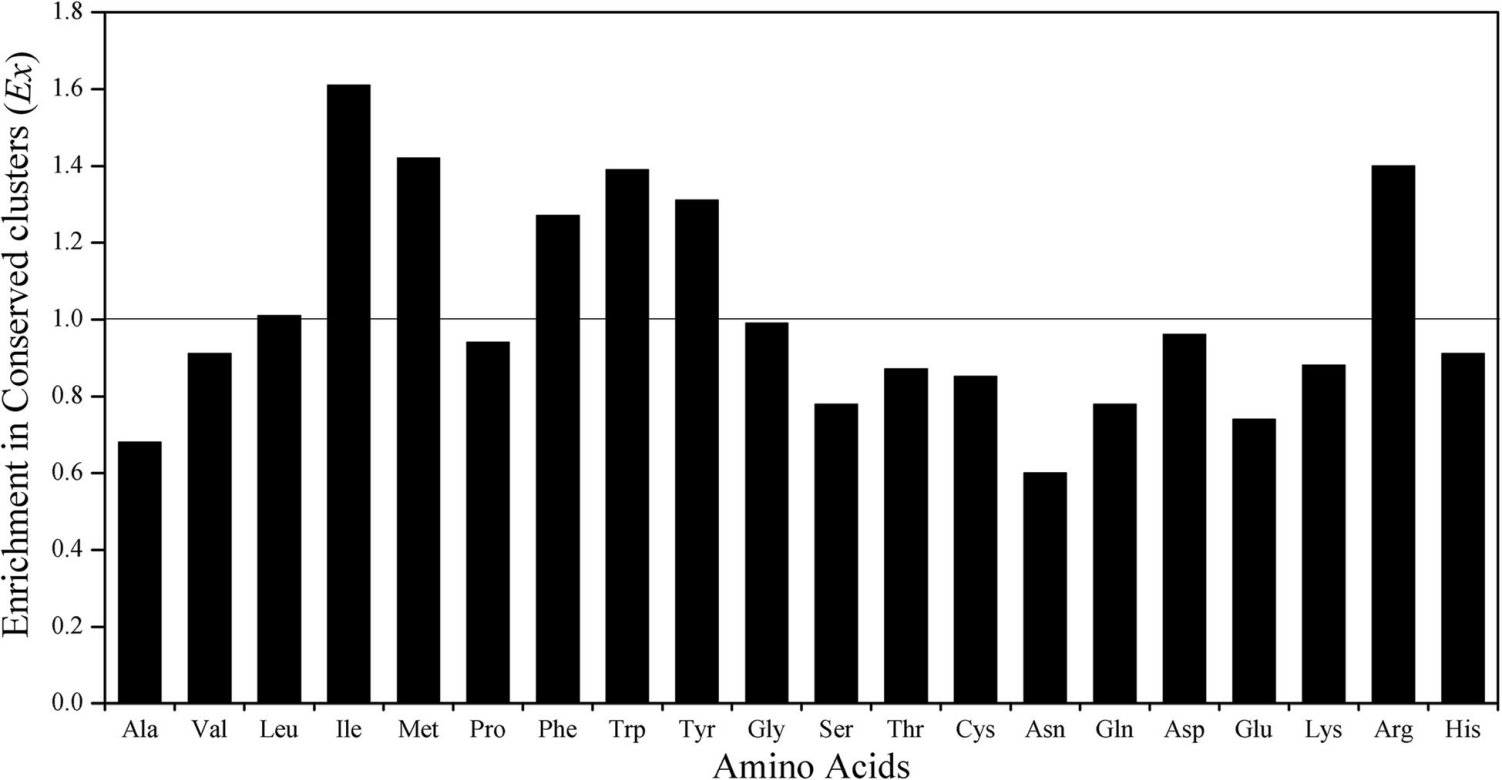
Relative enrichment of the 20 amino acid types within conserved clusters in protein-RNA interfaces

The result of preferences of amino acids in conserved residue clusters is given in Fig. 6. From Fig. 6, we can see that the hydrophobic (Leu, Ile, Met) and all the aromatic residues (Tyr, Phe, Trp) along with Arg are preferred in conserved interface clusters. Our previous study shows that all the three positively charged amino acids Arg, Lys and His are the most preferred ones in protein-RNA interfaces due to the negative electricity of RNAs [29], but here interestingly only Arg is preferred in the conserved subset of interface residues. Maybe this point can be explained by that Arg (2.64) has a significantly higher preference than Lys (1.78) and His (1.64) (This value greater than 1 indicates that the residue tends to appear on the interface) [29], which perhaps suggests Arg synergizes with other conserved residues in clusters to play an important role in function.

### Conserved residue clustering to discriminate the real interface from other random surface patches

**Fig. 7:**
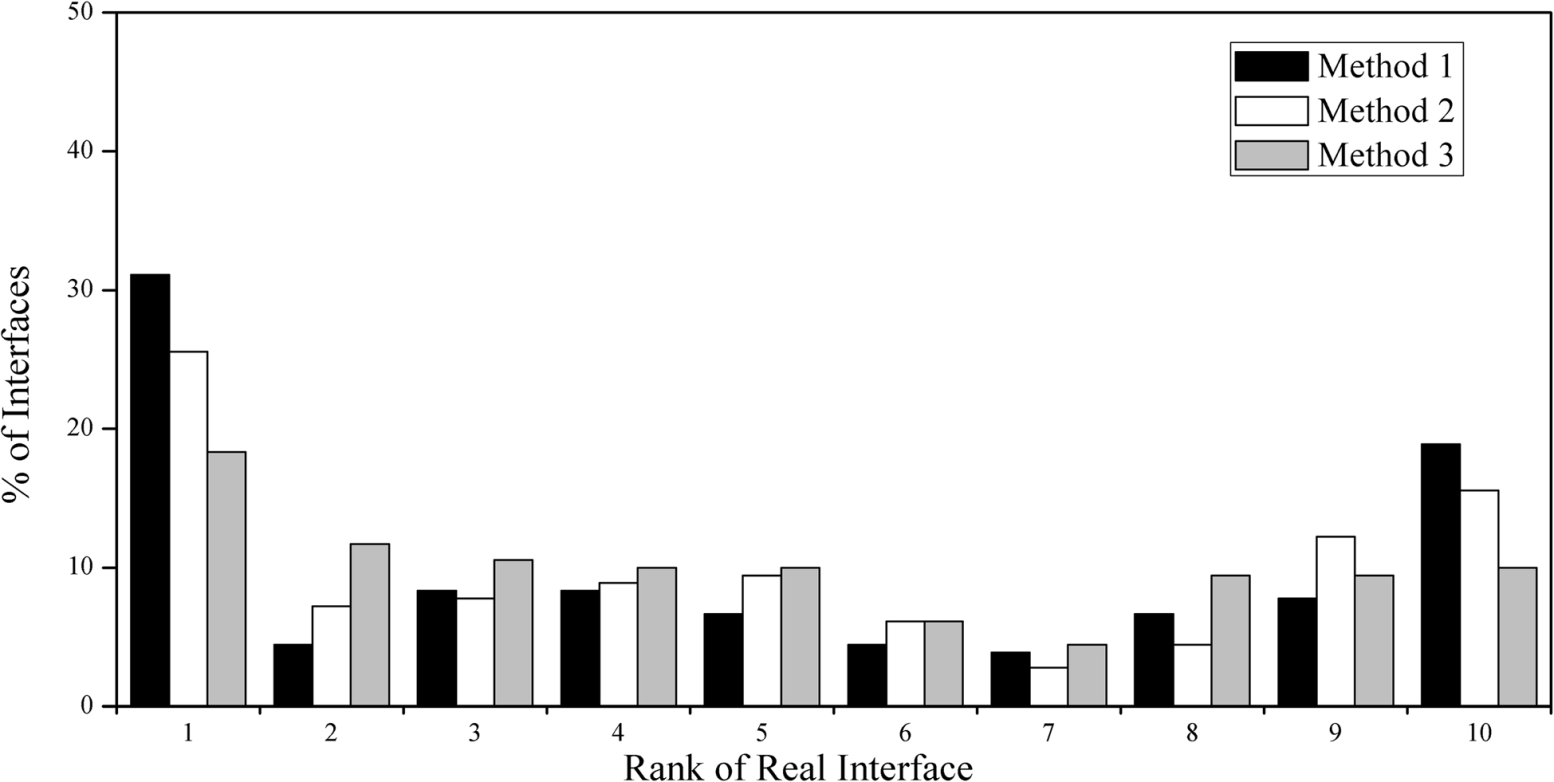
Ranking distribution of the real interface relative to all random surface patches according to the *ρ* value. Rank 1 means that the real interface is ranked the top 10% among it and all random surface patches, and rank 2 means the top 20%, etc. From method 1 to 3, the generated surface patch in a protein is more and more close to its own interface in size

To what extent can the clustering property of conserved residues be used to distinguish a real interface from the random surface patches? We compared *ρ* value of the interface region with those of the randomly generated surface patches for each protein. For each protein, 1000 random surface patches were produced using the method described in Materials and Methods. We ranked the real interface and the 1000 random surface patches in descending order of *ρ* for all proteins. A ranking of the real interface relative to the 1000 random surface patches was then calculated (on a scale of 1 to 10), and the results are shown in Fig. 7. Thus, for a ranking list, a rank 1 means that the real interface is ranked in the top 10% of all the randomly produced surface patches, and a rank 10 indicates the bottom 10%. Here we performed three different methods to generate surface patches. From method 1 to 3, the generated surface patches in a protein is more and more close to its own interface in size.

Fig. 7 shows the extent to which the coefficient *ρ* can distinguish the real interface region from all random surface patches. The similar ranking results are obtained by the three approaches of producing random surface patches. Out of 180 interfaces, there are 56 (31.1%), 46 (25.6%) and 33 (18.3%) interfaces that are ranked in the top 10% (i.e., rank 1) among all generated random surface patches by method 1 to 3, respectively. Therefore, to some extent, we can apply the clustering characteristics of interface conserved residues to distinguish the real interface from random surface patches.

**Fig. 8:**
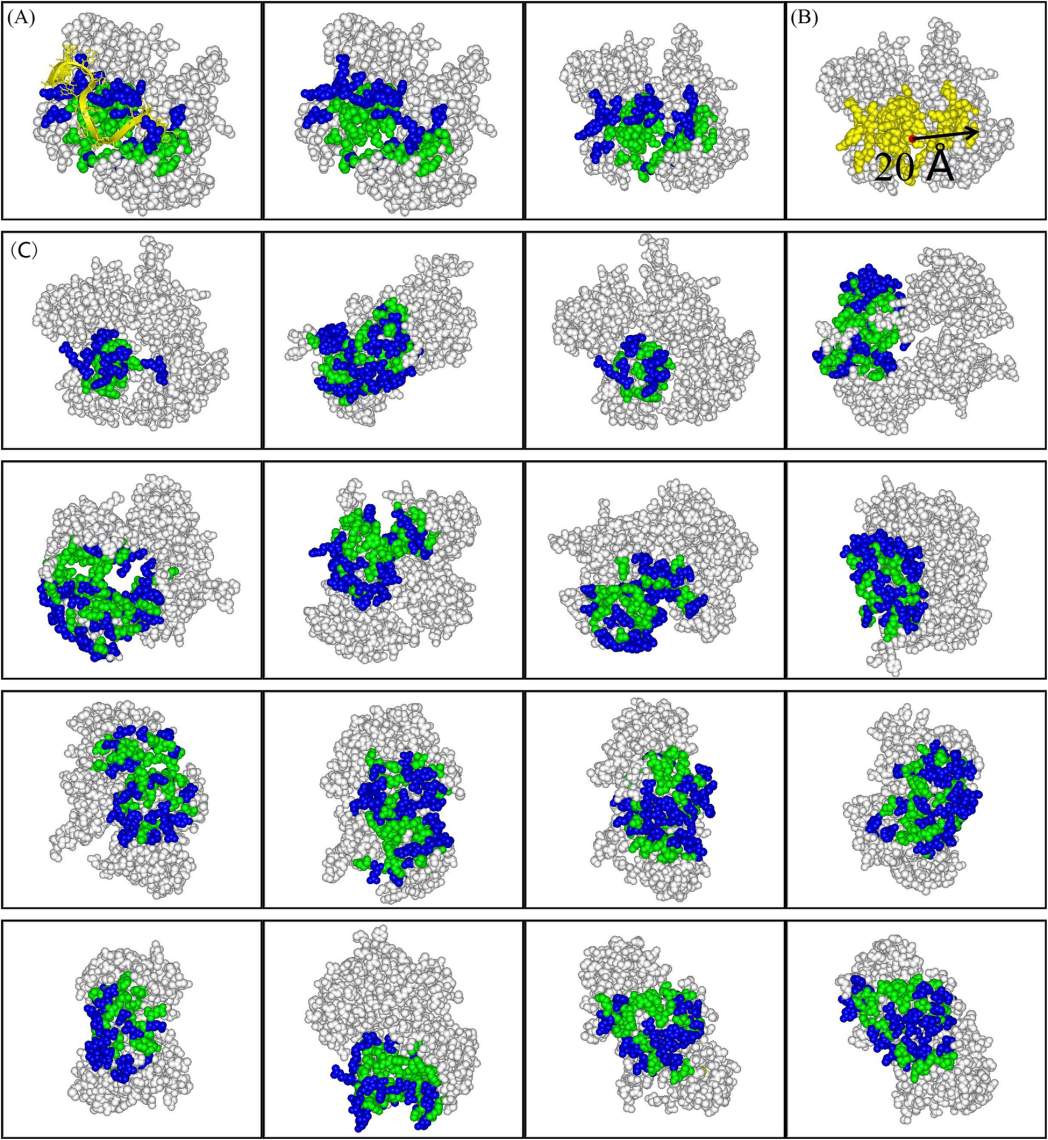
Comparison of the clustering of the conserved residues within the real interface and other random surface patches for the assembly of the SXL-UNR translation regulatory complex (PDB code: 4qqb). (**a**) Distributions of the conserved and the remaining residues at the real protein interface. In the first panel, RNA is in yellow and in the other two, it is removed to clearly show the clustering property of the conserved residues within the interface from different views. (**b**) The scheme of generating random surface patches with method 1 mentioned in materials and methods: a surface residue (represented by its Ca atom) is taken randomly as a center and then all the surface residues within 20 Å (half of the mean maximum distance 40 Å between any two atoms of all the interfaces) away from the center are chosen as belonging to the surface patch (yellow) with the taken residue as the center. (**c**) Distributions of the conserved and remaining residues at sixteen different random surface patches, where the conserved ones are relatively scattered over the random surface patch compared with those at the real interface. Protein is shown in CPK (grey), the conserved residues are in green and non-conserved ones in blue

Taking the SXL-UNR translation regulatory complex (PDB code: 4qqb) for example, we used the random surface patches generated by method 1 to measure the ability of *ρ* value to identify the real interface, because this method is closest to the actual condition when predicting the real interface (namely, we only know a universal interface size of protein-RNA complexes). Fig. 8 illustrates the clustering of conserved residues within the real interface in contrast with the distributions of conserved residues within randomly generated surface patches. From Fig. 8, different from the conserved residues in random surface patches which are distributed dispersedly over the regions, those in the interface regions are evidently clustered together.

### Extent of experimental hot spot residues occurring in conserved residue clusters

The analysis on the clustering of the conserved residues at the interface is instructive in identifying functionally important regions, because it is likely that hot spot residues are located in such clusters. For the 139 experimental alanine scanning mutagenesis data (involved in 41 protein-RNA complexes), we categorized them according to the seven amino acid classes, and the result shows they are distributed in all amino acid classes (Table 3). Additionally, we gave the plot of ΔΔG values versus sequence entropies for these residues (Figure S5 in supplementary materials). From Figure S5, the 139 interface residues have a wide range of sequence conservations and there is not an evident correlation between their ΔΔG values and sequence entropies. Then we performed the clustering analysis of the conserved interface residues on these proteins. Afterwards, the hot spot residues selected based on the experimental ΔΔG values of ≥1.0, ≥ 1.5 and ≥ 2.0 kcal/mol were mapped onto protein interfaces, respectively, and then the fractions of these residues occurring within the conserved residue clusters were calculated. The results are shown in Table S2 in supplementary materials.

**Table 3:** Distribution of 139 alanine scanned interface residues among the seven amino acid classes

Amino acid class	Num in entire dataset	Num of hot spot residues (ΔΔG ≥ 2.0 kcal/mol)	percentage of hot spot residues
Ala, Val, Leu, Ile, Met, Cys	3	1	33.33%
Gly, Ser, Thr	19	3	15.79%
Asp, Glu	16	1	6.25%
Asn, Gln	18	1	5.56%
Arg, Lys	49	10	20.41%
Pro, Phe, Tyr, Trp	27	16	59.26%
His	7	1	14.29%

From Table S2, out of the 75 residues with ΔΔG values ≥1.0 kcal/mol, there are 32 residues (42.7%) that occur within the conserved residue clusters. When further restricted to those residues with ΔΔG values ≥1.5 and ≥ 2.0 kcal/mol, the fraction increases to 46.5% (20/43) and 51.5% (17/33), respectively. Thus, there is a rising tendency that hot spot residues are located within the conserved clusters when a more stringent criterion is adopted. Additionally, interestingly, for the seven classes of amino acid residues, the three classes with the most largest percentages of hot spot residues (ΔΔG ≥ 2.0 kcal/mol) (see column 4 in Table 3) are the sixth class (Pro, Phe, Tyr, Trp) mainly containing aromatic residues, the first class (Ala, Val, Leu, Ile, Met, Cys) mainly containing hydrophobic residues, and the fifth class (Arg, Lys) positively charged residues, which are largely consistent with the preferred residues (Phe, Tyr, Trp, Leu, Ile, Met and Arg) in the conserved residue clusters. This further indicates that the preferred residues in the conserved interface clusters play an important role in protein-RNA structural stability and they can be used as candidates for the experimental alanine scanning mutagenesis study.

## Discussion and conclusions

This work mainly investigates the clustering extent of the conserved residues within protein-RNA interfaces. Four questions here are discussed: (1) probing evolutionary conservations of polypeptide sequences, (2) evaluating the clustering degree of the conserved residues within the interface, (3) analyzing the feasibility of using clustering degree (*ρ*) to distinguish the real interface from random surface regions, (4) exploring the extent of hot spot residues occurring in conserved residue clusters.

Based on the analyses above, regardless of which kinds of protein-RNA complexes are considered, the subset of conserved interface residues has a tendency to occur clustered together within the entire interface whatever stringent definitions of conserved residues are adopted. However, the clustering tendency of interface conserved residues is moderate (see Fig. 4). From the distribution of the *ρ* values for all interfaces (Figure S6 in supplementary materials). 73.9% (133/180) of interfaces have *ρ* values between 1.0 and 1.2, which can explain why the points in Fig. 4 are distributed near the diagonal. For protein-protein interactions, Guharoy et al. also found that the conserved interface residues are more spatially clustered relative to the entire interface residues, and the clustering property is not particularly high with almost 75% of the homodimeric interfaces and 50% of the heterocomplex interfaces having *ρ* values between 1.0 and 1.2, respectively [6]. The clustering of conserved interface residues may be more functionally important than a single, isolated conserved residue. The cooperativity between them in the cluster may form a network of interactions contributing to the stability of the complexes [30].

For the residues at interface, solvent exposed surface and interior regions, the former are more conserved than the middle, and the latter are the most conserved. This finding is in agreement with the previous study on protein-protein complexes [31]. The residues in protein interiors, providing stability to the folded polypeptide, are most conserved, and the interface residues are relatively conserved due to the evolutionary constraints for partner binding. At the interface, the aromatic residues Trp, Phe, and Tyr, along with Ile, Gly, Cys and Arg are more conserved. In interface conserved clusters, the hydrophobic (Leu, Ile, Met), all the aromatic (Tyr, Phe, Trp) and only one positively charged Arg residue are the preferred ones. As we know, the aromatic and Arg residues have important contributions to the stacking and ion-pi interactions with RNA bases respectively, which may explain the reason of their higher conservations. Based on our previous study [29, 32], the hydrophobic residues Leu, Ile and Met do not prefer to appear at protein-RNA interfaces, while interestingly they prefer to occur in the conserved interface clusters once they appear at interfaces. Considering the analysis results on hot spot residues that the hydrophobic residues have the second largest probability of being hot spot residues among the seven classes of amino acid residues, we think that the three kinds of preferred hydrophobic residues in conserved interface clusters maybe contribute an important role to protein-RNA binding free energy through their cooperative interactions with other residues in the conserved interface clusters. Additionally, the residue-nucleotide propensity potential obtained by us [29, 32] for protein-RNA interactions showed that Cys has a higher pairing preference with A and U, and Gly is relatively preferred by interfaces. For protein-RNA, protein-protein (homodimers and heterocomplexes) interactions, the common residues preferred in conserved clusters are Leu, Ile, Met, Tyr, Phe and Trp. Besides, Arg is preferred in protein-RNA, Val, Cys, Gly in homodimers, and Val, Cys, Gly, Asp in heterocomplexes. Thus, the charged residues do not tend to appear on the interface in homodimers, while the positively charged residue Asp is observed more as hot spot residues on the interface in heterocomplexes [33].

The clustering property of interface conserved residues can be utilized to distinguish the real interface from the random surface patches. In our result, 31% of the real interface regions are ranked in the top 10% of all random surface regions. Here we use the Z test to investigate whether it is of statistical significance that the clustering degree of the conserved residues within the real interface is relatively higher than that of the conserved ones in random surface region.

$$ Z=\frac{<\rho >-{\rho}_{\mathrm{int}}}{\sigma /\sqrt{n}} $$

where <*ρ* > is the mean value of *ρ* (with the standard deviation σ) for the *n* random surface patches in a protein and *ρ*_int_ is the *ρ* value for the real interface. For all the complexes, about 42.8% (77/180) of interfaces have conserved residues significantly more clustered compared with those present within surface patches (Z < 1.64, that means *ρ*_int_ of the real interface is not less than the *ρ* values of 95% random surface patches.). For protein-protein complexes, previous study shows that this value is 40% (49/121) and 38% (148/389) for the homodimers and heterocomplexes, respectively [6]. Therefore, for these interfaces, the clustered nature of the conserved residues can be used to differentiate well the true interface from surface patches.

For the hot spot residues (ΔΔG ≥ 2.0 kcal/mol), 51.5% of them are localized in the conserved residue clusters, and they are largely consistent with the preferred residue types in the conserved clusters, which indicates there exists the overlap to some extent between the conserved cluster and hot spot region, and the preferred residues can be used as targets for drug design and reference sites for experimental scanning mutagenesis studies. Now, the alanine scanning mutagenesis data are relatively limited, and with its increase, the further analysis can be performed and important findings may be achieved.

## Supplementary information

**Additional file 1: Table S1. **Dataset of 160 protein-RNA complexes (classified into five different function classes based on the type of RNA associated with the protein). **Table S2.** Location of experimental hot spots within the conserved residue clusters in protein interfaces. **Table S3.** Average entropy *<s>* of 20 types of amino acid residues in interior, interface and non-interface surface regions of protein-RNA complexes. **Table S4.** Values of the parameters indicating the clustering of conserved residues in individual interfaces. **Table S5.** Parameters describing the clustering of conserved interface residues in five classes of protein-RNA complexes. **Figure S1.** Conservation of the amino acid residues in five different protein-RNA complexes. Residue conservation is mapped at the protein surface with the color code provided at the bottom. Red stands for the maximum conservation (lowest *<s>*), and blue stands for the minimum conservation (highest *<s>*). The RNA backbone is shown in Stick and colored green. (A) The SXL-UNR translation regulatory complex (PDB code: 4qqb). (B) The prolyl-tRNA synthetase from thermus thermophilus complexed with tRNA (PDB code: 1h4q). (C) The ribosomal protein s8-rRNA complex (PDB code: 1i6u). (D) The bacterial protein-RNA toxin-antitoxin system (PDB code: 4rmo). (E) The human adenosine bound to dsRNA (PDB code: 5ed2). **Figure S2.** Distribution of the number of conserved interface residue sub-clusters as a function of the interface area in protein-RNA complexes. The x-axis labels mark the origin of the range in each column. Bins are of size 400 Å². **Figure S3**. Multiple clusters of evolutionary conserved residues in protein interfaces. (A) In the complex of prolyl-tRNA synthetase from thermus thermophilus complexed with tRNA (PDB code 1h4q, chain A with ρ = 1.17), the interface contains one well-clustered region of conserved residues. (B) In the complex of tRNA synthetase complexed with tRNA (PDB code 2du3, chain A with ρ = 1.19), the interface contains two regions of conserved residues. (C) Three conserved clusters in the interface of E. coli leucyl-tRNA synthetase with tRNA (PDB code 4arc, chain A with ρ = 1.19). Figures show the protein domains as CPK (green and blue for conserved and other residues), the RNA domains as Stick (yellow). **Figure S4.** Distribution of sub-cluster size (the number of interface residues in the conserved cluster). **Figure S5** Plot of ΔΔG values vs. sequence entropies for the 139 interface residues involved in 41 protein-RNA complexes for which experimental alanine scanning mutagenesis data are available. **Figure S6.** Percentage distribution of the *ρ* values for all protein-RNA interfaces.

## Data Availability

All data generated or analyzed during this study are included in this published article and its supplementary materials.

## Abbreviations

PDB: Protein data bank
RBP: RNA binding proteins
SASA: Solvent accessible surface area
