# Supplementary material for: Analyses on clustering of the conserved residues at protein-RNA interfaces and its application in binding site identification
Source: BMC Bioinformatics. 2020 Feb 17;21:57. doi: 10.1186/s12859-020-3398-9 (PMC7027071; doi:10.1186/s12859-020-3398-9)
Supplement: Supplementary file 1 — Additional file 1: Table S1. Dataset of 160 protein-RNA complexes (classified into five different function classes based on the type of RNA associated with the protein). Table S2. Location of experimental hot spots within the conserved residue clusters in protein interfaces. Table S3. Average entropy of 20 types of amino acid residues in interior, interface and non-interface surface regions of protein-RNA complexes. Table S4. Values of the parameters indicating the clustering of conserved residues in individual interfaces. Table S5. Parameters describing the clustering of conserved interface residues in five classes of protein-RNA complexes. Figure S1. Conservation of the amino acid residues in five different protein-RNA complexes. Residue conservation is mapped at the protein surface with the color code provided at the bottom. Red stands for the maximum conservation (lowest ), and blue stands for the minimum conservation (highest ). The RNA backbone is shown in Stick and colored green. (A) The SXL-UNR translation regulatory complex (PDB code: 4qqb). (B) The prolyl-tRNA synthetase from thermus thermophilus complexed with tRNA (PDB code: 1h4q). (C) The ribosomal protein s8-rRNA complex (PDB code: 1i6u). (D) The bacterial protein-RNA toxin-antitoxin system (PDB code: 4rmo). (E) The human adenosine bound to dsRNA (PDB code: 5ed2). Figure S2. Distribution of the number of conserved interface residue sub-clusters as a function of the interface area in protein-RNA complexes. The x-axis labels mark the origin of the range in each column. Bins are of size 400 Å². Figure S3. Multiple clusters of evolutionary conserved residues in protein interfaces. (A) In the complex of prolyl-tRNA synthetase from thermus thermophilus complexed with tRNA (PDB code 1h4q, chain A with ρ = 1.17), the interface contains one well-clustered region of conserved residues. (B) In the complex of tRNA synthetase complexed with tRNA (PDB code 2du3, chain A with ρ = 1.19), the interface contai [file 12859_2020_3398_MOESM1_ESM.doc]

**Supplementary Materials**

**Analyses on clustering of the conserved residues at protein-RNA interfaces and its application in binding site identification**

Zhen Yang^†^, Xueqing Deng^†^, Yang Liu, Weikang Gong, Chunhua Li^*^

College of Life Science and Bioengineering, Beijing University of Technology, Beijing 100124, China

*All correspondence should be addressed to Chunhua Li (E-mail: [chunhuali@bjut.edu.cn)](mailto:chunhuali@bjut.edu.cn))

^†^ Zhen Yang and Xueqing Deng contributed equally to this work.

Table S1: Dataset of 160 protein-RNA complexes (classified into five different function classes based on the type of RNA associated with the protein).

| PDB ID^a^ | Composition^b^ | Res^c^ | Pro Chid^d^ | Chain Length^e^ | Aligned  Sequences^f^ | *<s>*^g^ |
| --- | --- | --- | --- | --- | --- | --- |
| 1. **mRNA (8)** | | | | | | |
| 1S03 | Ribosomal protein S8 | 2.70 | H | 127 | 3828 | 0.94 |
| 4F02 | EIF4G with PABP and poly(A) | 2.00 | A | 175 | 724 | 0.83 |
| 4QOZ | HISTONE mRNA binding protein, and 3'hexo ternary | 2.30 | B | 277 | 69 | 0.82 |
|  |  |  | C | 73 | 231 | 1.11 |
| 4QQB | the assembly of the SXL-UNR translation regulatory complex | 2.80 | A | 169 | 133 | 0.84 |
|  |  |  | B | 169 | 133 | 0.84 |
| 4TUW | Drosophila binding protein with mRNA | 2.90 | A | 71 | 216 | 1.00 |
| 5F5H | Complex with OX40 HEXA-loop RNA motif | 2.23 | A | 148 | 48 | 0.54 |
| 5M0I | Complex with she2p and the ASH1 mRNA | 2.41 | A | 225 | 19 | 0.81 |
| 5SUP | mRNA export factors | 2.60 | A | 383 | 274 | 0.51 |
| Average |  |  |  |  |  | 0.82 |
| 1. **tRNA (53)** | | | | | | |
| 1B23 | E. coli Cysteinyl-tRNA synthetase | 2.60 | P | 405 | 3430 | 0.69 |
| 1C0A | E. coli Aspartyl-tRNA synthetase | 2.40 | A | 585 | 4394 | 0.94 |
| 1EFW | T. thermophilus-tRNA synthetase | 3.00 | A | 580 | 4881 | 0.97 |
| 1GAX | T. thermophilus Valyl-tRNA synthetase | 2.90 | A | 862 | 2103 | 1.07 |
| 1H3E | T. thermophilus Tyrosyl-tRNA synthetase | 2.90 | A | 427 | 2118 | 1.08 |
| 1H4Q | T. Thermophilus Prolyl-tRNA | 3.0 | A | 465 | 2518 | 0.97 |
| 1IL2 | E. coli Aspartyl-tRNA synthetase | 2.60 | A | 585 | 4404 | 0.94 |
| 1J1U | M. jannaschii Tyrosyl-tRNA synthetase | 1.95 | A | 299 | 146 | 0.83 |
| 1K8W | E. coli Pseudouridine synthase | 1.85 | A | 304 | 1188 | 1.00 |
| 1N78 | T. thermophilus Glutamyl-tRNA synthetase | 2.10 | A | 468 | 513 | 1.06 |
| 1QF6 | E. coli Threonyl-tRNA synthetase | 2.90 | A | 641 | 3049 | 0.95 |
| 1QTQ | E. Coli Glutaminyl-tRNA synthetase | 2.25 | A | 529 | 3490 | 0.93 |
| 1R3E | tRNA Pseudouridine synthase | 2.10 | A | 305 | 61 | 1.07 |
| 1SER | T. thermophilusSeryl-tRNA synthetase | 2.90 | A | 372 | 90 | 0.94 |
| 1TTT | Phe-tRNA, elongation factor | 2.70 | A | 405 | 3430 | 0.69 |
| 1U0B | E.coli Cysteinyl-tRNA synthetase | 2.30 | B | 461 | 3642 | 1.10 |
| 1VFG | tRNA nucleotidyl transferase | 2.80 | A | 342 | 10 | 0.63 |
| 2AB4 | Pseudouridine 55 synthase | 2.40 | A | 300 | 54 | 1.06 |
| 2AZX | Human Tryptophanyl-tRNA synthetase | 2.80 | A | 387 | 710 | 0.86 |
| 2B3J | tRNA Adenosine Deaminase | 2.00 | A | 151 | 5354 | 1.06 |
| 2CSX | Methionyl-tRNA synthetase | 2.70 | A | 464 | 2452 | 1.04 |
| 2DU3 | O-Phosphoseryl-tRNA synthetase | 2.60 | A | 534 | 90 | 0.84 |
| 2DXI | Glutamyl-tRNA synthetase | 2.20 | A | 468 | 523 | 1.06 |
| 2FK6 | Rnase Z/tRNA | 2.90 | A | 307 | 1021 | 0.93 |
| 2FMT | Methionyl-tRNAfmet transformylase | 2.80 | A | 314 | 3507 | 1.08 |
| 2GJW | RNA recognition and cleavage | 2.85 | A | 308 | 8 | 0.74 |
| 2HVY | H/ACA box RNP from Pyrococcus furiosus | 2.30 | A | 320 | 417 | 0.96 |
|  |  |  | B | 74 | 45 | 0.84 |
|  |  |  | C | 53 | 339 | 1.05 |
|  |  |  | D | 121 | 332 | 0.77 |
| 2RFK | Archaeal H/ACA ribonucleo protein | 2.87 | A | 334 | 393 | 0.97 |
|  |  |  | C | 74 | 50 | 0.91 |
| 2ZUE | Arginyl-tRNA synthetase | 2.00 | A | 628 | 31 | 0.55 |
| 2ZZM | ATRM5 and tRNA | 2.65 | A | 333 | 12 | 0.66 |
| 3ADD | O-Phosphoseryl-tRNA Kinase | 2.40 | A | 251 | 17 | 0.75 |
| 3AKZ | Glutamyl- tRNA synthetase | 2.90 | A | 463 | 142 | 1.08 |
| 3AM1 | O-Phosphoseryl-tRNA Kinase | 2.40 | A | 236 | 17 | 0.75 |
| 3AMT | the Tias-tRNA(Ile2)-ATP complex | 2.90 | A | 417 | 85 | 0.95 |
| 3BT7 | E. coli 5-Methyluridine tRMA | 2.43 | A | 369 | 697 | 0.88 |
| 3FOZ | E. coli Isopentenyl-tRNA transferase | 2.50 | A | 305 | 1752 | 0.98 |
| 3KFU | the Transamidosome | 3.00 | C | 377 | 361 | 1.00 |
|  |  |  | H | 467 | 4778 | 0.92 |
|  |  |  | J | 83 | 18 | 0.71 |
| 3LWR | H/ACA RNP bound to a substrate RNA | 2.20 | A | 317 | 419 | 0.97 |
|  |  |  | B | 53 | 336 | 1.03 |
|  |  |  | C | 120 | 351 | 0.81 |
| 3OVB | CCA-adding enzyme | 1.95 | A | 441 | 7 | 0.55 |
| 3VJR | Peptidyl-tRNA Hydrolase | 2.40 | A | 193 | 1694 | 1.02 |
| 4ARC | E. coli Leucyl-tRNA synthetase | 2.00 | A | 813 | 3984 | 1.08 |
| 4GCW | Rnase Z with Precursor tRNA | 3.00 | A | 307 | 1039 | 0.92 |
| 4JXX | E. coli Glutaminyl-tRNA synthetase | 2.30 | A | 536 | 3490 | 0.93 |
| 4KR6 | A 4-Thiouridine synthetase - RNA | 2.85 | A | 388 | 41 | 0.90 |
| 4QEI | Glyrs Captured | 2.88 | A | 562 | 610 | 0.94 |
| 4WC2 | tRNA Nucleotidyl transferase | 2.80 | A | 367 | 9 | 0.56 |
| 4YCO | E. coli Dihydrouridine synthase | 2.10 | A | 311 | 492 | 0.90 |
| 4ZDO | T325S mutant of Human Sepsecs | 2.65 | A | 445 | 206 | 0.92 |
| 5AXM | THG1 like protein | 2.21 | A | 213 | 47 | 0.87 |
| 5CCB | Human MLA58 Methyltransferase | 2.00 | A | 282 | 331 | 1.00 |
|  |  |  | B | 371 | 64 | 0.68 |
| 5D0A | Epoxyqueuosine Reductase | 2.10 | A | 379 | 631 | 0.80 |
| 5HR7 | E. coli in vitro Transcribed tRNA | 2.40 | A | 360 | 1374 | 0.92 |
| 5UD5 | Pyrrolysyl-tRNA synthetase | 2.35 | A | 86 | 19 | 0.64 |
| Average |  |  |  |  |  | 0.90 |
| 1. **rRNA (23)** | | | | | | |
| 1DFU | E.coli ribosomal protein L25 | 1.80 | P | 94 | 730 | 1.00 |
| 1DK1 | A key element of the ribosome assembly | 2.80 | A | 86 | 5193 | 1.01 |
| 1FEU | Ribosomal protein TL5 | 2.30 | A | 185 | 9 | 0.43 |
| 1I6U | Ribosomal protein S8/rRNA | 2.60 | A | 129 | 583 | 0.93 |
| 1MJI | Ribosomal protein L5/5S rRNA | 2.50 | A | 180 | 4885 | 0.84 |
| 1MMS | Ribosomal protein L11-RNA | 2.57 | A | 133 | 4191 | 0.85 |
| 1MZP | L1 Protuberance in the ribosome | 2.65 | A | 217 | 21 | 0.71 |
| 1QA6 | A conserved ribosomal protein-RNA | 2.80 | A | 67 | 3833 | 0.85 |
| 1RLG | Archaeal SRNP intiation | 2.70 | A | 113 | 409 | 0.77 |
| 1Y39 | Co-evolution of protein and RNA | 2.80 | A | 74 | 3868 | 0.83 |
| 3FTF | A. Aeolicus KSGA | 2.80 | A | 246 | 18 | 0.75 |
| 3IEV | Era in complex with MGGNP | 1.90 | A | 302 | 489 | 0.76 |
| 3NMU | Substrate-bound halfmer box C/D RNP | 2.73 | A | 366 | 88 | 0.92 |
|  |  |  | F | 227 | 544 | 0.86 |
|  |  |  | G | 121 | 339 | 0.77 |
| 3NVI | N-Terminal Runcated NOP56/58 | 2.71 | A | 247 | 88 | 0.92 |
|  |  |  | B | 121 | 339 | 0.77 |
| 3UMY | Mutant ribosomal protein T217A TTHL1 | 1.90 | A | 228 | 4233 | 0.81 |
| 4BW0 | the L7AE class of proteins | 2.33 | B | 117 | 397 | 0.79 |
| 4IFD | AN 11-subunit Eukaryotic Exosome complex | 2.81 | A | 300 | 135 | 0.83 |
|  |  |  | B | 242 | 55 | 0.61 |
|  |  |  | D | 223 | 36 | 0.78 |
|  |  |  | I | 230 | 42 | 0.92 |
| 4LGT | Catalytic domain of RLUB | 1.30 | A | 251 | 1058 | 0.97 |
| 5C0Y | RRP6 catalytic domain bound to poly(u) RNA | 2.10 | A | 386 | 179 | 1.07 |
| 5D8H | Methanococcus with antibiotic Thiostrepton | 2.80 | C | 158 | 316 | 0.92 |
| 5G4U | Association of three two-K-turn units | 2.65 | C | 120 | 397 | 0.79 |
| 5G4V | Association of four two-K-turn units | 2.87 | C | 117 | 397 | 0.79 |
| 5WZH | APUM23-GGAAUUGACGG | 2.51 | A | 533 | 63 | 0.76 |
| Average |  |  |  |  |  | 0.83 |
| 1. **Viral RNA (7)** | | | | | | |
| 2GIC | Virus nucleocapsid-RNA | 2.92 | A | 421 | 17 | 0.75 |
| 2XD0 | A processed non-coding RNA | 3.00 | A | 162 | 79 | 0.82 |
| 3RW6 | Nuclear RNA export factor TAP | 2.30 | A | 245 | 250 | 0.84 |
| 4H5P | Rift valley fever virus | 2.15 | A | 244 | 41 | 0.84 |
| 4JNG | Schmallenberg virus Nucleoprotein | 2.12 | A | 226 | 16 | 0.72 |
| 4RMO | CPTIN type III toxin-antitoxin system | 2.20 | A | 155 | 16 | 0.62 |
| 4WSB | Bat Influenza a Polymerase | 2.65 | A | 699 | 21 | 0.23 |
|  |  |  | C | 734 | 15 | 0.25 |
| Average |  |  |  |  |  | 0.63 |
| 1. **Other RNA (69)** | | | | | | |
| 1A9N | Spliceosomal U2B''-U2A' protein | 2.38 | A | 162 | 435 | 0.89 |
|  |  |  | B | 94 | 519 | 0.83 |
| 1CVJ | POLY(A)-binding protein | 2.60 | A | 169 | 733 | 0.87 |
| 1EC6 | K-Homology RNA-binding domain | 2.40 | A | 87 | 117 | 0.95 |
| 1HQ1 | A universally conserved protein | 1.52 | A | 76 | 3652 | 1.01 |
| 1JBS | Ribotoxin restrictocin and A 29-mer SRD RNA | 1.97 | A | 149 | 8 | 0.53 |
| 1JID | Human SRP19 | 1.80 | A | 114 | 232 | 0.81 |
| 1LNG | SRP19-7S.S SRP RNA | 2.30 | A | 87 | 42 | 1.05 |
| 1M5O | State stabilization by A catalytic RNA | 2.20 | C | 92 | 478 | 0.87 |
| 1OOA | NF-KB(P50)2 | 2.45 | A | 313 | 75 | 0.82 |
| 1R9F | P19 with 19-BP Small interfering RNA | 1.85 | A | 121 | 8 | 0.43 |
| 1RPU | CIRV P19 bound to siRNA | 2.50 | A | 138 | 11 | 0.46 |
| 1URN | U1A mutant/RNA | 1.92 | A | 96 | 485 | 0.84 |
| 1WSU | C-terminal domain of elongation factor | 2.30 | A | 124 | 19 | 0.94 |
| 2ANR | NOVA-1 KH1/KH2 domain Tandem with 25NT RNA hairpin | 1.94 | A | 155 | 95 | 0.76 |
| 2IX1 | Rnase II D209N mutant | 2.74 | A | 643 | 321 | 0.74 |
| 2J0S | Exon junction complex | 2.21 | A | 391 | 617 | 0.76 |
|  |  |  | T | 44 | 57 | 1.01 |
| 2JEA | A 9-subunit Archaeal Exosome | 2.33 | A | 274 | 57 | 0.92 |
|  |  |  | B | 234 | 259 | 0.72 |
| 2NUG | Rnase III from AQUIFEX AEOLICUS | 1.70 | A | 216 | 10 | 0.70 |
| 2OIH | Hepatitis Delta virus gemonic ribozyme | 2.40 | A | 95 | 478 | 0.87 |
| 2OZB | A Human PRP31-15.5K-U4 snRNA | 2.60 | A | 126 | 348 | 0.62 |
|  |  |  | B | 239 | 274 | 0.79 |
| 2R8S | Specific synthetic FAB | 1.95 | H | 218 | 909 | 0.78 |
|  |  |  | L | 214 | 274 | 0.54 |
| 2V3C | the SRP54-SRP19-7S.S SRP RNA | 2.50 | C | 403 | 337 | 0.98 |
| 2XLK | the Csy4-crRNA complex | 1.80 | A | 188 | 179 | 1.06 |
| 2Y8Y | Crispr Endoribonuclease CSE3 | 1.44 | A | 201 | 13 | 0.49 |
| 2ZKO | Structural basis for dsRNA | 1.70 | A | 70 | 34 | 0.37 |
| 3A6P | Exportin-5:RANGTP:PRE-miRNA | 2.92 | A | 1072 | 89 | 0.70 |
|  |  |  | C | 170 | 585 | 0.76 |
| 3EGZ | Tetracycline Aptamer and artificial riboswitch | 2.20 | A | 91 | 481 | 0.87 |
| 3KS8 | Reston Ebolavirus VP35 RNA binding domain | 2.40 | A | 122 | 9 | 0.33 |
| 3SIU | HPRP31-15.5K-U4ATAC 5' | 2.63 | A | 125 | 518 | 0.62 |
|  |  |  | B | 235 | 426 | 0.81 |
| 3SNP | Iron regulatory protein | 2.80 | A | 850 | 2855 | 0.90 |
| 3TRZ | Mouse LIN28A LET-7D MicroRNA | 2.90 | A | 130 | 120 | 0.94 |
| 3TS2 | Mouse LIN28A LET-7C MicroRNA | 2.01 | A | 135 | 120 | 0.94 |
| 3V7E | SAM-I Riboswitch Aptamer | 2.80 | A | 81 | 492 | 0.88 |
| 3WBM | Protein-RNA complex | 2.00 | A | 86 | 242 | 0.83 |
| 4C8Y | CAS6 (TTHA0078) substrate mimic complex | 1.80 | A | 238 | 19 | 0.53 |
| 4C9D | CAS6 (TTHB231) product complex | 3.00 | A | 262 | 6 | 0.46 |
| 4IG8 | Cytosolic double-stranded RNA | 2.70 | A | 338 | 323 | 1.07 |
| 4J39 | P19 with double-helical 19MER RNA | 1.70 | A | 122 | 8 | 0.43 |
| 4JGN | RNA silencing suppressor P19 | 1.86 | A | 125 | 8 | 0.43 |
| 4K4Z | Coxsackievirus B3 Polymerase elongation | 2.17 | A | 462 | 123 | 0.74 |
| 4KNQ | 1NT-5'-overhanging double | 1.82 | A | 122 | 8 | 0.43 |
| 4KRE | Human Argonaute-1 | 1.75 | A | 799 | 110 | 0.79 |
| 4KRF | Human Argonaute-1 Let-7 complex | 2.10 | A | 817 | 110 | 0.79 |
| 4KZD | Fluorophore and FAB | 2.19 | H | 225 | 869 | 0.72 |
| 4M30 | Rnase III complexed with double-stranded RNA | 2.50 | A | 220 | 495 | 0.70 |
| 4M4O | the Aptamer mine-Lysozyme complex | 2.00 | A | 129 | 10 | 1.02 |
| 4M59 | the Pentatricopeptide repeat protein PPR10 | 2.46 | A | 687 | 58 | 0.57 |
| 4N0T | the U6 small nuclear ribonucleoprotein | 1.70 | A | 363 | 18 | 0.85 |
| 4O26 | TRBD domain of TERT and the CR4/5 OF TR | 3.00 | A | 240 | 49 | 0.67 |
| 4OOG | Yeast Rnase III (RNT1P) | 2.50 | A | 109 | 19 | 0.87 |
|  |  |  | C | 261 | 30 | 0.84 |
| 4QIK | the ROQ domain of Human ROQUIN | 1.90 | A | 302 | 96 | 0.80 |
| 4RWN | the PRE-Reactive state of Porcine OAS1 | 2.00 | A | 349 | 308 | 1.08 |
| 4U7U | RNA-guided immune Cascade complex | 3.00 | A | 480 | 28 | 0.89 |
|  |  |  | B | 157 | 13 | 0.72 |
|  |  |  | D | 191 | 26 | 0.90 |
|  |  |  | E | 357 | 46 | 0.80 |
|  |  |  | K | 218 | 42 | 0.94 |
| 4Z4D | Human Argonaute2 | 1.60 | A | 803 | 120 | 0.84 |
| 4ZLD | Human ROQUIN-2 ROQ domain | 1.60 | A | 151 | 47 | 0.56 |
| 4ZT0 | Catalytically-active CAS9 | 2.90 | A | 1280 | 82 | 0.90 |
| 5AOR | MLE RNA ADP ALF4 COMPLEX | 2.08 | A | 1009 | 166 | 0.91 |
| 5AOX | Human ALU RNA Retrotransposition | 2.04 | A | 76 | 129 | 0.88 |
|  |  |  | B | 86 | 150 | 0.96 |
| 5ED1 | Human Adenosine Deaminase acting on dsRN | 2.77 | A | 396 | 171 | 0.96 |
| 5ED2 | Human Adenosine Deaminase acting on dsRNA | 2.95 | A | 385 | 171 | 0.96 |
| 5F5F | ROQUIN ROQ domain | 3.00 | A | 152 | 354 | 0.63 |
| 5F9F | RIG-I HELICASE-RD | 2.60 | A | 673 | 134 | 0.79 |
| 5GUH | SILKWORM PIWI-CLADE ARGONAUTE | 2.40 | A | 759 | 105 | 1.08 |
| 5I9F | Designed Pentatricopeptide repeat protein | 2.19 | A | 388 | 1131 | 1.36 |
| 5JC7 | Chicken MDA5 WITH 5'P 24-MER dsRNA | 2.75 | A | 641 | 107 | 0.80 |
| 5JJI | RHO Transcription termination factor | 2.60 | A | 405 | 3104 | 0.88 |
| 5L2L | NAB2 ZN Fingers 5-7 bound to A11G RNA | 1.55 | A | 72 | 87 | 0.88 |
| 5T7B | Argonaute-2 - 5'-(E)-VINYLPHOSPHONATE 2'-O-METHYL | 2.53 | A | 802 | 115 | 0.81 |
| 5UDZ | Human LIN28A with MicroRNA | 2.00 | A | 139 | 323 | 0.98 |
| Average |  |  |  |  |  | 0.80 |

^a^PDB ID of protein-RNA complexes.

^b^Composition of the complexes

^c^Resolution of the X-ray structures.

^d^Chain ids of the protein subunit in the PDB entry.

^e^Length of the protein chains.

^f^Multiple sequence alignments of the protein chains obtained from the UniRef90 database.

^g^Average entropy calculated for the entire polypeptide chain.

Table S2. Location of experimental hot spots within the conserved residue clusters in protein interfaces.

| PDB code & chain id | Protein name^a^ | Statistics on clustering of conserved residues in the interface | | | Num of experimental hot spots^b^ (num occurring within conserved clusters) | | |
| --- | --- | --- | --- | --- | --- | --- | --- |
|  |  | *M_s,cons_* | *M_s,int_* | *ρ* | ≥ 1 | ≥ 1.5 | ≥ 2 |
| 1ASY_A | Yeast aspartyl-tRNA synthetase | 0.084 | 0.077 | 1.09 | 13 (7) | 3 (2) | 2 (2) |
| 1AUD_A | Human U1A protein | 0.093 | 0.083 | 1.12 | 6 (4) | 4 (3) | 4 (3) |
| 1C9S_L | RNA-binding attenuation protein | 0.077 | 0.068 | 1.13 | 4 (1) | 4 (1) | 4 (1) |
| 1JBS_A | Ribotoxin restrictocin | 0.078 | 0.069 | 1.13 | 2 (2) | 1 (1) | - |
| 1QFQ_B | Bacteriophage Lambda N-protein | 0.095 | 0.091 | 1.04 | 1 (1) | 1 (1) | 1 (1) |
| 1U0B_B | Cysteinyl-tRNA synthetase | 0.054 | 0.064 | 0.84 | 1 (0) | 1 (0) | - |
| 1YVP_A | Ro autoantigen | 0.078 | 0.081 | 0.96 | - | - | - |
| 2BX2_L | E. Coli Rnase E Catalytic Domain | 0.089 | 0.088 | 1.01 | - | - | - |
| 2ERR_A | Human alternative splicing factor Fox-1 | 0.087 | 0.083 | 1.05 | 4 (2) | 4 (2) | 3 (2) |
| 2IX1_A | RNase II D209N mutant | - | 0.078 | - | - | - | - |
| 2PJP_A | Elongation factor SelB from E.coli | 0.101 | 0.099 | 1.02 | 3 (1) | 2 (1) | 2 (1) |
| 2Y8W_A | CRISPR endoribonuclease Cse3 | 0.084 | 0.112 | 0.75 | 1 (0) | - | - |
| 2ZKO_A | NS1 protein of human influenza virus A | 0.074 | 0.069 | 1.07 | 2 (1) | - | - |
| 2ZZM_A | Anticodon loop modification | - | 0.088 | - | - | - | - |
| 3EQT_A | Human LGP2 C-terminal domain | 0.123 | 0.131 | 0.94 | 1 (0) | 1 (0) | - |
| 3K5Y_A | FBF-2/gld-1 FBEa complex | 0.064 | 0.056 | 1.14 | - | - | - |
| 3MOJ_B | Bacillus subtilis YxiN protein | 0.059 | 0.061 | 0.97 | 2 (0) | - | - |
| 3QSU_A | Staphylococcus aureus Hfq | 0.089 | 0.088 | 1.01 | 1 (1) | 1 (1) | - |
| 3RW6_A | nucleocytoplasmic transport | 0.061 | 0.063 | 0.97 | - | - | - |
| 3U4M_A | ribosomal protein tthl1 | 0.139 | 0.101 | 1.38 | 1 (1) | 1 (1) | 1 (1) |
| 4ALP_A | Lin28b Cold shock domain | - | 0.154 | - | 1 (0) | 1 (0) | 1 (0) |
| 4ED5_A | two N-terminal RRM domains of HuR | 0.099 | 0.105 | 0.94 | 1 (0) | - | - |
| 4HT8_A | E coli Hfq | 0.088 | 0.091 | 0.97 | 1 (0) | 1 (0) | 1 (0) |
| 4NGD_A | Human Dicer Platform-PAZ-Connector Helix cassette | 0.087 | 0.064 | 1.36 | 1 (0) | 1 (0) | 1 (0) |
| 4NKU_A | Cid1 protein | 0.107 | 0.101 | 1.06 | - | - | - |
| 4OOG_C | yeast RNase III | 0.086 | 0.084 | 1.02 | 3 (2) | - | - |
| 4QVC_D | E coli Hfq | 0.099 | 0.074 | 1.34 | 2 (0) | 1 (0) | 1 (0) |
| 4QVD_D | E coli Hfq | 0.085 | 0.081 | 1.05 | - | - | - |
| 4R3I_A | YTHDC1 YTH domain | 0.159 | 0.148 | 1.07 | 1 (0) | 1 (0) | 1 (0) |
| 4R8I_A | Mirror-Image RNA Oligonucleotide Aptamer | 0.120 | 0.090 | 1.33 | 3 (1) | 2 (1) | 2 (1) |
| 4RCJ_A | YTHDF1 YTH domain | 0.074 | 0.071 | 1.04 | 2 (2) | 1 (1) | 1 (1) |
| 5DET_A | Human RBPMS | 0.049 | 0.048 | 1.02 | 2 (1) | 2 (1) | 2 (1) |
| 5ELK_A | mouse Unkempt zinc fingers 4-6 | 0.078 | 0.069 | 1.13 | 2 (1) | 2 (1) | - |
| 5GXH_A | Gemin5 WD40 domain | 0.080 | 0.055 | 1.45 | 4 (1) | 3 (0) | 2 (0) |
| 5H1K_A | double-WD40 repeat domain of Gemin | 0.078 | 0.077 | 1.01 | 6 (3) | 5 (3) | 4 (3) |
| 5JBJ_A | chicken LGP2 | 0.110 | 0.097 | 1.13 | - | - | - |
| 5M0J_J | mRNA-transport complex | 0.068 | 0.077 | 0.88 | - | - | - |
| 5M3H_A | Bat influenza A/H17N10 polymerase | 0.085 | 0.074 | 1.15 | - | - | - |
| 5UDZ_A | Human LIN28A | 0.080 | 0.072 | 1.11 | - | - | - |
| 5WWW_A | E3 ubiquitin-protein | 0.145 | 0.138 | 1.05 | 1 (0) | - | - |
| 5WWX_A | human RNA-binding E3 ubiquitin-protein | 0.084 | 0.074 | 1.14 | 2 (0) | - | - |
| Overall extent of localization of experimental hot spots within conserved residue clusters | | | | | 75 (32) | 43 (20) | 33 (17) |

^a^ The names of the protein components in complexes with the chain id of that component given along with the PDB code in the first column.

^b^ Hot spot residues are defined in three criteria with ΔΔG values ≥ 1.0, or ≥ 1.5, or ≥ 2.0 kcal/mol. Components for which experimental alanine scanning data are not available are marked with '-'.

Table S3: Average entropy *<s>* of 20 types of amino acid residues in interior, interface and non-interface surface regions of protein-RNA complexes.

|  | protein-RNA complexes | | |
| --- | --- | --- | --- |
| Amino acid residues | Interior | Interface | Non-interface surface |
| Ala | 0.52 | 0.85 | 1.27 |
| Val | 0.24 | 0.56 | 0.82 |
| Leu | 0.24 | 0.53 | 0.73 |
| Ile | 0.20 | 0.32 | 0.68 |
| Met | 0.31 | 0.64 | 0.78 |
| Pro | 0.35 | 0.70 | 1.03 |
| Phe | 0.40 | 0.39 | 0.72 |
| Trp | 0.18 | 0.38 | 0.58 |
| Tyr | 0.31 | 0.46 | 0.85 |
| Gly | 0.36 | 0.41 | 0.77 |
| Ser | 0.56 | 0.73 | 1.17 |
| Thr | 0.52 | 0.70 | 1.13 |
| Cys | 0.41 | 0.42 | 0.83 |
| Asn | 0.49 | 0.88 | 1.34 |
| Gln | 0.40 | 0.92 | 1.41 |
| Asp | 0.19 | 0.78 | 1.03 |
| Glu | 0.36 | 0.84 | 1.27 |
| Lys | 0.22 | 0.65 | 1.25 |
| Arg | 0.26 | 0.52 | 1.03 |
| His | 0.49 | 0.71 | 1.26 |

**Table S4. Values of the parameters indicating the clustering of conserved residues in individual interfaces.**

| PDB code | *<s>_int_*^a^  (num of aligned sequences) | Num of interface, conserved interface residues | *M_s_* | | *ρ* | Num of sub-clusters |
| --- | --- | --- | --- | --- | --- | --- |
|  |  |  | Conserved residues | All interface residues |  |  |
| 1a9n_A | 0.97 (435) | 4,2 | 0.171 | 0.178 | 0.96 | 1 |
| 1a9n_B | 0.77 (519) | 37,22 | 0.095 | 0.089 | 1.07 | 1 |
| 1b23_P | 0.48 (3430) | 53,37 | 0.065 | 0.061 | 1.07 | 1 |
| 1c0a_A | 0.79 (4394) | 73,41 | 0.055 | 0.049 | 1.12 | 2 |
| 1cvj_A | 0.42 (733) | 46,33 | 0.071 | 0.070 | 1.02 | 1 |
| 1dfu_P | 0.61 (730) | 26,14 | 0.099 | 0.094 | 1.06 | 1 |
| 1dk1_A | 0.88 (5193) | 36,19 | 0.085 | 0.074 | 1.15 | 1 |
| 1ec6_A | 0.71 (117) | 30,15 | 0.097 | 0.087 | 1.12 | 1 |
| 1efw_A | 1.13 (4881) | 42,19 | 0.075 | 0.062 | 1.20 | 1 |
| 1feu_A | 0.38 (9) | 26,17 | 0.093 | 0.082 | 1.14 | 1 |
| 1gax_A | 0.97 (2103) | 100,53 | 0.040 | 0.038 | 1.07 | 2 |
| 1h3e_A | 1.36 (2118) | 49,21 | 0.067 | 0.060 | 1.11 | 3 |
| 1h4q_A | 1.30 (2518) | 10,3 | 0.243 | 0.137 | 1.77 | 1 |
| 1hq1_A | 1.14 (3652) | 20,10 | 0.122 | 0.108 | 1.13 | 1 |
| 1i6u_A | 0.91 (583) | 30,15 | 0.098 | 0.084 | 1.17 | 1 |
| 1il2_A | 0.87 (4404) | 72,40 | 0.052 | 0.049 | 1.07 | 1 |
| 1j1u_A | 0.81 (146) | 21,11 | 0.123 | 0.086 | 1.42 | 1 |
| 1jbs_A | 0.45 (8) | 27,12 | 0.085 | 0.083 | 1.03 | 1 |
| 1jid_A | 0.61 (232) | 26,16 | 0.095 | 0.088 | 1.08 | 1 |
| 1k8w_A | 0.28 (1188) | 48,38 | 0.074 | 0.074 | 1.01 | 1 |
| 1lng_A | 1.06 (42) | 30,16 | 0.097 | 0.084 | 1.16 | 1 |
| 1m5o_C | 0.74 (478) | 29,18 | 0.096 | 0.092 | 1.04 | 1 |
| 1mji_A | 0.78 (4885) | 30,17 | 0.082 | 0.082 | 1.00 | 1 |
| 1mms_A | 0.87 (4191) | 36,17 | 0.092 | 0.079 | 1.16 | 1 |
| 1mzp_A | 0.57 (21) | 38,22 | 0.069 | 0.070 | 1.00 | 1 |
| 1n78_A | 0.85 (513) | 85,47 | 0.052 | 0.046 | 1.13 | 1 |
| 1ooa_A | 0.34 (75) | 28,20 | 0.085 | 0.079 | 1.06 | 1 |
| 1qa6_A | 0.78 (3833) | 28,17 | 0.098 | 0.090 | 1.09 | 1 |
| 1qf6_A | 0.56 (3049) | 77,46 | 0.050 | 0.048 | 1.05 | 1 |
| 1qtq_A | 0.81 (3490) | 82,44 | 0.048 | 0.046 | 1.03 | 1 |
| 1r3e_A | 0.36 (61) | 47,33 | 0.076 | 0.073 | 1.04 | 1 |
| 1r9f_A | 0.27 (8) | 29,21 | 0.087 | 0.083 | 1.05 | 1 |
| 1rlg_A | 0.58 (409) | 18,9 | 0.102 | 0.104 | 0.98 | 1 |
| 1rpu_A | 0.41 (11) | 18,11 | 0.080 | 0.081 | 0.99 | 1 |
| 1s03_H | 0.77 (3828) | 29,16 | 0.096 | 0.087 | 1.11 | 1 |
| 1ser_A | 0.49 (90) | 8,4 | 0.115 | 0.126 | 0.91 | 1 |
| 1ttt_A | 0.49 (3430) | 50,34 | 0.067 | 0.062 | 1.09 | 1 |
| 1u0b_B | 0.83 (3642) | 85,42 | 0.056 | 0.051 | 1.09 | 2 |
| 1urn_A | 0.81 (485) | 28,16 | 0.099 | 0.092 | 1.07 | 1 |
| 1vfg_A | 0.66 (10) | 16,8 | 0.073 | 0.079 | 0.92 | 3 |
| 1wsu_A | 0.17 (19) | 13,9 | 0.119 | 0.117 | 1.02 | 1 |
| 1y39_A | 0.76 (3868) | 30,18 | 0.098 | 0.088 | 1.12 | 1 |
| 2ab4_A | 0.36 (54) | 49,32 | 0.076 | 0.073 | 1.04 | 1 |
| 2anr_A | 0.43 (95) | 21,14 | 0.116 | 0.106 | 1.09 | 1 |
| 2azx_A | 0.75 (710) | 23,14 | 0.109 | 0.085 | 1.29 | 1 |
| 2b3j_A | 0.67 (5354) | 28,18 | 0.100 | 0.092 | 1.09 | 1 |
| 2csx_A | 0.86 (2452) | 40,21 | 0.060 | 0.061 | 0.99 | 1 |
| 2du3_A | 0.47 (90) | 29,17 | 0.088 | 0.074 | 1.19 | 2 |
| 2dxi_A | 0.86 (523) | 88,48 | 0.052 | 0.046 | 1.13 | 1 |
| 2fk6_A | 1.04 (1021) | 23,12 | 0.075 | 0.069 | 1.09 | 2 |
| 2fmt_A | 0.81 (3507) | 43,22 | 0.070 | 0.064 | 1.08 | 1 |
| 2gic_A | 0.51 (17) | 36,25 | 0.073 | 0.067 | 1.08 | 1 |
| 2gjw_A | 0.23 (8) | 22,16 | 0.081 | 0.081 | 1.01 | 2 |
| 2hvy_A | 0.77 (417) | 45,27 | 0.071 | 0.067 | 1.05 | 1 |
| 2hvy_C | 0.90 (339) | 9,5 | 0.155 | 0.115 | 1.35 | 1 |
| 2hvy_D | 0.53 (332) | 19,10 | 0.099 | 0.104 | 0.95 | 1 |
| 2ix1_A | 0.27 (296) | 80,56 | 0.051 | 0.050 | 1.02 | 2 |
| 2j0s_A | 0.43 (617) | 28,21 | 0.093 | 0.082 | 1.13 | 1 |
| 2j0s_T | 0.30 (57) | 2,1 | - | 0.107 | - | - |
| 2jea_A | 0.28 (57) | 9,6 | 0.115 | 0.115 | 1.00 | 1 |
| 2jea_B | 0.60 (259) | 21,10 | 0.082 | 0.078 | 1.05 | 2 |
| 2nug_A | 0.46 (10) | 28,17 | 0.082 | 0.076 | 1.09 | 1 |
| 2oih_A | 0.84 (478) | 31,18 | 0.094 | 0.090 | 1.04 | 1 |
| 2ozb_A | 0.30 (348) | 19,15 | 0.104 | 0.103 | 1.02 | 1 |
| 2ozb_B | 0.57 (274) | 23,14 | 0.091 | 0.088 | 1.04 | 1 |
| 2r8s_H | 1.75 (909) | 24,9 | 0.122 | 0.100 | 1.21 | 1 |
| 2r8s_L | 1.39 (274) | 18,9 | 0.114 | 0.093 | 1.22 | 1 |
| 2rfk_A | 1.17 (393) | 14,6 | 0.110 | 0.097 | 1.14 | 1 |
| 2v3c_C | 0.99 (337) | 54,29 | 0.060 | 0.056 | 1.07 | 2 |
| 2xd0_A | 0.51 (79) | 34,20 | 0.077 | 0.078 | 0.99 | 1 |
| 2xlk_A | 0.96 (179) | 33,16 | 0.077 | 0.074 | 1.04 | 1 |
| 2y8y_A | 0.41 (13) | 36,27 | 0.080 | 0.076 | 1.05 | 1 |
| 2zko_A | 0.06 (34) | 12,9 | 0.093 | 0.098 | 0.95 | 1 |
| 2zue_A | 0.47 (31) | 76,41 | 0.052 | 0.049 | 1.05 | 3 |
| 2zzm_A | 0.51 (12) | 77,46 | 0.056 | 0.053 | 1.05 | 2 |
| 3a6p_A | 0.67 (57) | 72,43 | 0.044 | 0.044 | 0.99 | 2 |
| 3a6p_C | 1.34 (333) | 3,2 | 0.186 | 0.211 | 0.88 | 1 |
| 3add_A | 0.76 (17) | 50,29 | 0.051 | 0.049 | 1.03 | 2 |
| 3akz_A | 0.85 (142) | 85,47 | 0.050 | 0.047 | 1.08 | 1 |
| 3am1_A | 0.56 (17) | 38,22 | 0.063 | 0.057 | 1.11 | 2 |
| 3amt_A | 0.64 (85) | 65,38 | 0.064 | 0.057 | 1.13 | 2 |
| 3bt7_A | 0.29 (697) | 42,30 | 0.076 | 0.071 | 1.08 | 1 |
| 3egz_A | 0.83 (481) | 30,16 | 0.100 | 0.092 | 1.08 | 1 |
| 3foz_A | 0.54 (1752) | 64,45 | 0.069 | 0.065 | 1.06 | 1 |
| 3ftf_A | 0.59 (18) | 18,11 | 0.073 | 0.076 | 0.96 | 1 |
| 3iev_A | 0.79 (489) | 38,23 | 0.094 | 0.081 | 1.15 | 1 |
| 3kfu_C | 1.31 (361) | 30,14 | 0.084 | 0.078 | 1.07 | 1 |
| 3ks8_A | 0.13 (9) | 18,6 | 0.109 | 0.097 | 1.12 | 1 |
| 3lwr_A | 0.82 (419) | 47,25 | 0.071 | 0.068 | 1.05 | 1 |
| 3lwr_B | 0.63 (336) | 7,5 | 0.153 | 0.151 | 1.02 | 1 |
| 3lwr_C | 0.65 (351) | 20,10 | 0.099 | 0.104 | 0.95 | 1 |
| 3nmu_A | 0.28 (88) | 37,26 | 0.091 | 0.085 | 1.07 | 1 |
| 3nmu_F | 1.28 (544) | 2,1 | - | 0.131 | - | - |
| 3nmu_G | 0.67 (339) | 25,13 | 0.095 | 0.089 | 1.07 | 1 |
| 3nvi_A | 0.30 (88) | 39,29 | 0.094 | 0.086 | 1.09 | 1 |
| 3nvi_B | 0.56 (339) | 19,10 | 0.098 | 0.103 | 0.95 | 1 |
| 3ovb_A | 0.30 (7) | 52,38 | 0.060 | 0.054 | 1.11 | 2 |
| 3rw6_A | 0.96 (250) | 44,22 | 0.066 | 0.064 | 1.03 | 1 |
| 3siu_A | 0.30 (348) | 19,15 | 0.105 | 0.103 | 1.02 | 1 |
| 3siu_B | 0.59 (267) | 22,13 | 0.094 | 0.091 | 1.03 | 1 |
| 3snp_A | 0.37 (2855) | 60,47 | 0.059 | 0.057 | 1.04 | 1 |
| 3trz_A | 0.54 (120) | 26,19 | 0.103 | 0.097 | 1.06 | 1 |
| 3ts2_A | 0.51 (120) | 28,20 | 0.099 | 0.093 | 1.07 | 1 |
| 3umy_A | 0.77 (4233) | 48,28 | 0.070 | 0.067 | 1.04 | 1 |
| 3v7e_A | 0.34 (492) | 16,10 | 0.106 | 0.116 | 0.91 | 1 |
| 3vjr_A | 0.86 (1694) | 20,10 | 0.096 | 0.087 | 1.11 | 1 |
| 3wbm_A | 0.34 (242) | 12,8 | 0.109 | 0.119 | 0.92 | 1 |
| 4arc_A | 0.89 (3984) | 77,41 | 0.051 | 0.043 | 1.19 | 3 |
| 4bw0_B | 0.56 (397) | 18,10 | 0.098 | 0.105 | 0.94 | 1 |
| 4c8y_A | 0.49 (19) | 40,25 | 0.080 | 0.077 | 1.04 | 1 |
| 4c9d_A | 0.32 (6) | 38,26 | 0.081 | 0.081 | 1.01 | 1 |
| 4f02_A | 0.43 (724) | 44,33 | 0.073 | 0.071 | 1.03 | 1 |
| 4gcw_A | 0.82 (1039) | 33,18 | 0.068 | 0.063 | 1.07 | 2 |
| 4h5p_A | 0.30 (41) | 39,26 | 0.075 | 0.078 | 0.96 | 1 |
| 4ifd_A | 0.24 (135) | 6,4 | 0.122 | 0.123 | 0.99 | 1 |
| 4ifd_B | 0.77 (55) | 10,6 | 0.059 | 0.068 | 0.88 | 3 |
| 4ig8_A | 1.17 (323) | 25,13 | 0.082 | 0.076 | 1.08 | 1 |
| 4j39_A | 0.21 (8) | 22,18 | 0.088 | 0.082 | 1.08 | 1 |
| 4jgn_A | 0.33 (8) | 29,21 | 0.082 | 0.076 | 1.07 | 1 |
| 4jng_A | 0.48 (16) | 44,28 | 0.071 | 0.068 | 1.06 | 1 |
| 4jxx_A | 0.84 (3490) | 86,46 | 0.047 | 0.046 | 1.02 | 1 |
| 4k4z_A | 0.43 (123) | 48,34 | 0.073 | 0.066 | 1.10 | 1 |
| 4knq_A | 0.27 (8) | 26,21 | 0.084 | 0.079 | 1.07 | 1 |
| 4kr6_A | 0.94 (41) | 8,4 | 0.109 | 0.087 | 1.25 | 1 |
| 4kre_A | 0.31 (110) | 77,49 | 0.053 | 0.048 | 1.09 | 3 |
| 4krf_A | 0.31 (110) | 81,49 | 0.052 | 0.048 | 1.09 | 3 |
| 4kzd_H | 2.18 (869) | 17,8 | 0.104 | 0.111 | 0.94 | 1 |
| 4lgt_A | 0.53 (1058) | 58,39 | 0.071 | 0.067 | 1.05 | 1 |
| 4m30_A | 0.51 (495) | 23,14 | 0.083 | 0.083 | 1.01 | 1 |
| 4m4o_A | 0.82 (10) | 9,5 | 0.119 | 0.131 | 0.91 | 1 |
| 4m59_A | 0.21 (58) | 33,20 | 0.076 | 0.077 | 0.99 | 1 |
| 4n0t_A | 0.66 (18) | 79,47 | 0.065 | 0.055 | 1.18 | 1 |
| 4o26_A | 0.68 (49) | 37,22 | 0.082 | 0.079 | 1.03 | 1 |
| 4oog_A | 0.35 (19) | 5,4 | 0.160 | 0.136 | 1.18 | 1 |
| 4oog_C | 0.59 (30) | 45,29 | 0.062 | 0.060 | 1.02 | 1 |
| 4qei_A | 0.66 (610) | 54,30 | 0.061 | 0.056 | 1.09 | 1 |
| 4qik_A | 0.26 (96) | 7,3 | 0.097 | 0.115 | 0.84 | 1 |
| 4qoz_B | 0.54 (69) | 47,30 | 0.065 | 0.060 | 1.08 | 1 |
| 4qoz_C | 0.49 (231) | 23,14 | 0.088 | 0.093 | 0.94 | 1 |
| 4qqb_A | 1.81 (133) | 1,1 | - | - | - | - |
| 4qqb_B | 0.61 (133) | 60,36 | 0.076 | 0.068 | 1.12 | 1 |
| 4rmo_A | 0.60 (16) | 36,21 | 0.082 | 0.075 | 1.08 | 1 |
| 4rwn_A | 1.25 (308) | 28,14 | 0.086 | 0.073 | 1.18 | 1 |
| 4tuw_A | 0.43 (216) | 21,13 | 0.086 | 0.090 | 0.96 | 1 |
| 4u7u_A | 0.49 (28) | 4,3 | 0.232 | 0.204 | 1.14 | 1 |
| 4u7u_D | 0.47 (26) | 42,28 | 0.089 | 0.077 | 1.15 | 1 |
| 4u7u_E | 0.25 (46) | 41,30 | 0.072 | 0.066 | 1.08 | 1 |
| 4u7u_K | 0.35 (42) | 42,30 | 0.077 | 0.076 | 1.02 | 1 |
| 4wc2_A | 0.33 (9) | 44,27 | 0.048 | 0.050 | 0.97 | 1 |
| 4wsb_A | 0.14 (21) | 48,34 | 0.073 | 0.069 | 1.07 | 1 |
| 4wsb_C | 0.10 (15) | 10,10 | 0.114 | 0.114 | 1.00 | 1 |
| 4yco_A | 0.65 (492) | 40,23 | 0.073 | 0.065 | 1.13 | 1 |
| 4z4d_A | 0.36 (120) | 92,59 | 0.049 | 0.045 | 1.09 | 2 |
| 4zdo_A | 1.43 (206) | 22,10 | 0.061 | 0.067 | 0.91 | 2 |
| 4zld_A | 0.36 (47) | 33,24 | 0.084 | 0.080 | 1.05 | 1 |
| 4zt0_A | 0.66 (82) | 167,101 | 0.043 | 0.041 | 1.06 | 1 |
| 5aor_A | 0.41 (166) | 68,48 | 0.060 | 0.057 | 1.06 | 1 |
| 5aox_A | 0.98 (129) | 16,6 | 0.117 | 0.104 | 1.12 | 1 |
| 5aox_B | 0.94 (150) | 23,13 | 0.088 | 0.085 | 1.03 | 1 |
| 5axm_A | 0.80 (47) | 33,17 | 0.097 | 0.084 | 1.16 | 1 |
| 5c0y_A | 1.27 (179) | 13,8 | 0.089 | 0.100 | 0.89 | 1 |
| 5ccb_B | 0.23 (64) | 58,40 | 0.056 | 0.051 | 1.11 | 1 |
| 5d0a_A | 0.82 (631) | 16,9 | 0.075 | 0.084 | 0.90 | 1 |
| 5d8h_C | 1.15 (316) | 33,18 | 0.088 | 0.080 | 1.11 | 1 |
| 5ed1_A | 0.53 (171) | 32,19 | 0.094 | 0.077 | 1.21 | 1 |
| 5ed2_A | 0.50 (171) | 35,20 | 0.093 | 0.077 | 1.22 | 1 |
| 5f5f_A | 0.31 (60) | 26,19 | 0.086 | 0.084 | 1.02 | 1 |
| 5f5h_A | 0.33 (48) | 27,19 | 0.090 | 0.084 | 1.06 | 1 |
| 5f9f_A | 0.58 (94) | 63,40 | 0.059 | 0.058 | 1.01 | 1 |
| 5g4u_C | 0.61 (397) | 15,8 | 0.107 | 0.114 | 0.93 | 1 |
| 5g4v_C | 0.58 (397) | 16,9 | 0.103 | 0.114 | 0.90 | 1 |
| 5guh_A | 0.47 (105) | 46,31 | 0.051 | 0.051 | 1.00 | 2 |
| 5hr7_A | 0.73 (1374) | 57,34 | 0.067 | 0.062 | 1.09 | 1 |
| 5i9f_A | 1.50 (1131) | 61,36 | 0.056 | 0.056 | 1.00 | 1 |
| 5jc7_A | 0.73 (107) | 32,19 | 0.059 | 0.057 | 1.04 | 1 |
| 5jji_A | 0.19 (3104) | 6,5 | 0.166 | 0.179 | 0.92 | 1 |
| 5l2l_A | 0.67 (87) | 18,12 | 0.102 | 0.097 | 1.05 | 1 |
| 5m0i_A | 0.00 (19) | 3,3 | 0.210 | 0.210 | 1.00 | 1 |
| 5sup_A | 0.56 (274) | 27,18 | 0.095 | 0.086 | 1.10 | 1 |
| 5t7b_A | 0.38 (115) | 77,47 | 0.053 | 0.048 | 1.11 | 2 |
| 5ud5_A | 0.57 (19) | 30,16 | 0.096 | 0.093 | 1.03 | 1 |
| 5udz_A | 0.51 (119) | 30,21 | 0.095 | 0.090 | 1.05 | 1 |
| 5wzh_A | 0.56 (63) | 65,37 | 0.057 | 0.057 | 1.00 | 1 |

For protein-RNA complexes, the calculation is carried out separately in the interface regions of the proteins forming the complexes - the chain id of the component is mentioned after the PDB code.

For a few interfaces containing only a single or no conserved residues, the clustering could not be carried out and this is indicated with a dash ('-') in the appropriate column.

^a^Average sequence entropy calculated over all interface residues. The number of homologous sequences is indicated.

Table S5. Parameters describing the clustering of conserved interface residues in five classes of protein-RNA complexes.

|  | Average | | | Num of interfaces | |
| --- | --- | --- | --- | --- | --- |
| Interface type | *M_s,int_* | *M_s,cons_* | *ρ* | Total | with *M_s,cons_* greater than  *M_s,int_* |
| with tRNA | 0.071 (0.02) | 0.078 (0.03) | 1.09 (0.13) | 58 | 50 |
|  | 0.069 (0.03) | 0.081 (0.04) | 1.15 (0.25) | 57 | 50 |
|  | 0.066 (0.02) | 0.080 (0.02) | 1.24 (0.32) | 51 | 44 |
| with rRNA | 0.087 (0.02) | 0.090 (0.01) | 1.03 (0.08) | 26 | 14 |
|  | 0.087 (0.02) | 0.090 (0.02) | 1.04 (0.12) | 25 | 13 |
|  | 0.088 (0.02) | 0.100 (0.03) | 1.14 (0.32) | 23 | 14 |
| with mRNA | 0.095 (0.04) | 0.098 (0.04) | 1.04 (0.06) | 8 | 5 |
|  | 0.095 (0.04) | 0.101 (0.04) | 1.08 (0.10) | 8 | 5 |
|  | 0.095 (0.04) | 0.100 (0.04) | 1.07 (0.29) | 8 | 4 |
| with viral RNA | 0.077 (0.02) | 0.080 (0.01) | 1.03 (0.04) | 8 | 5 |
|  | 0.071 (0.01) | 0.074 (0.01) | 1.03 (0.11) | 7 | 4 |
|  | 0.071 (0.01) | 0.073 (0.01) | 1.02 (0.12) | 7 | 4 |
| with other RNA | 0.088 (0.03) | 0.092 (0.03) | 1.05 (0.07) | 80 | 66 |
|  | 0.084 (0.03) | 0.093 (0.03) | 1.11 (0.13) | 75 | 63 |
|  | 0.082 (0.03) | 0.088 (0.03) | 1.11 (0.22) | 61 | 48 |

Three sets of values are provided, corresponding to three different criteria to define the subset of conserved interface residues (see Materials and Methods).

^a^ Standard deviations are in parentheses.

^b^ A smaller number of interfaces is reported in the second and third rows of data where because of the use of a more stringent condition of conservation, some interfaces, with 0 or 1 conserved residue, get excluded from consideration.


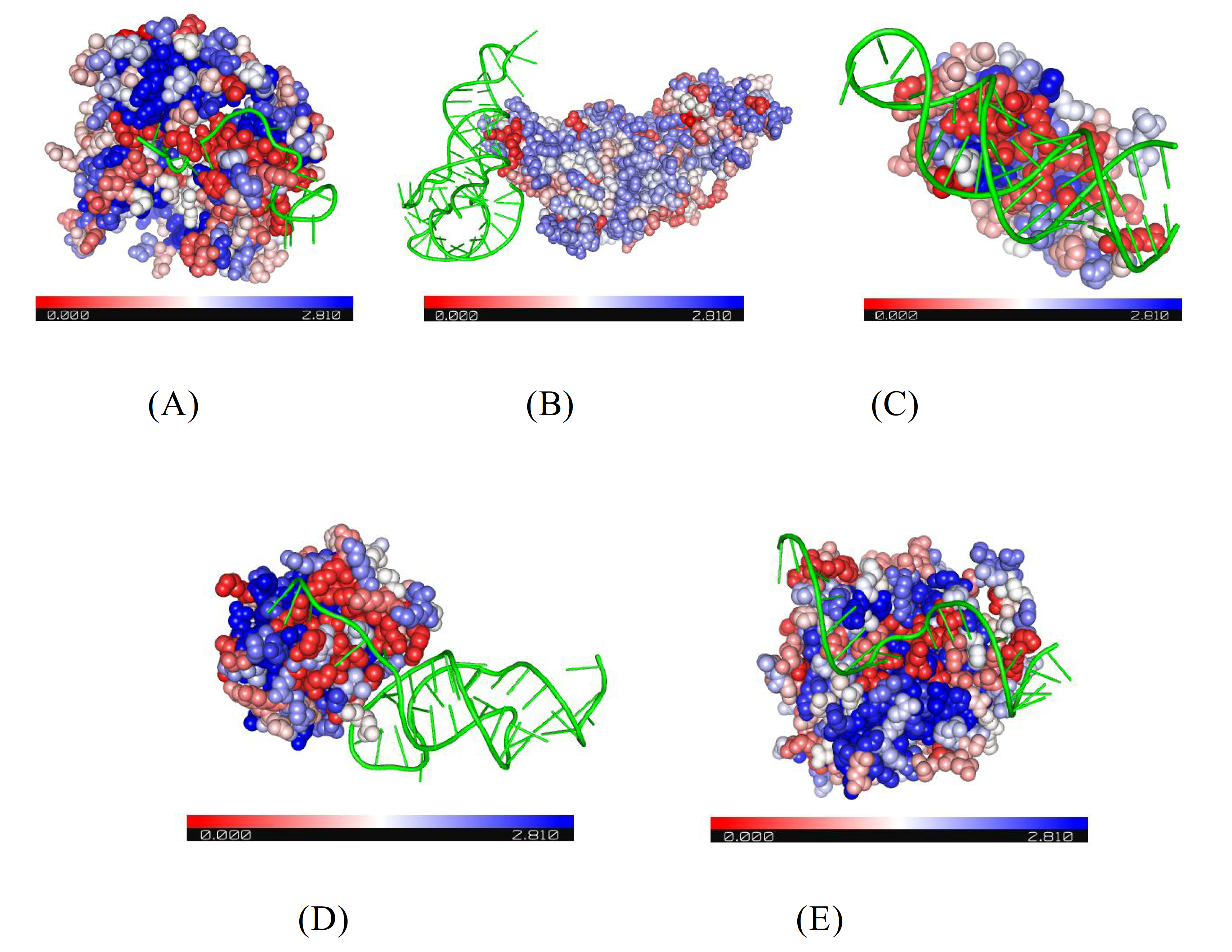


Figure S1. Conservation of the amino acid residues in five different protein-RNA complexes. Residue conservation is mapped at the protein surface with the color code provided at the bottom. Red stands for the maximum conservation (lowest *<s>*), and blue stands for the minimum conservation (highest *<s>*). The RNA backbone is shown in Stick and colored green. (A) The SXL-UNR translation regulatory complex (PDB code: 4qqb). (B) The prolyl-tRNA synthetase from thermus thermophilus complexed with tRNA (PDB code: 1h4q). (C) The ribosomal protein s8-rRNA complex (PDB code: 1i6u). (D) The bacterial protein-RNA toxin-antitoxin system (PDB code: 4rmo). (E) The human adenosine bound to dsRNA (PDB code: 5ed2).





Figure S2. Distribution of the number of conserved interface residue sub-clusters as a function of the interface area in protein-RNA complexes. The x-axis labels mark the origin of the range in each column. Bins are of size 400 Å².


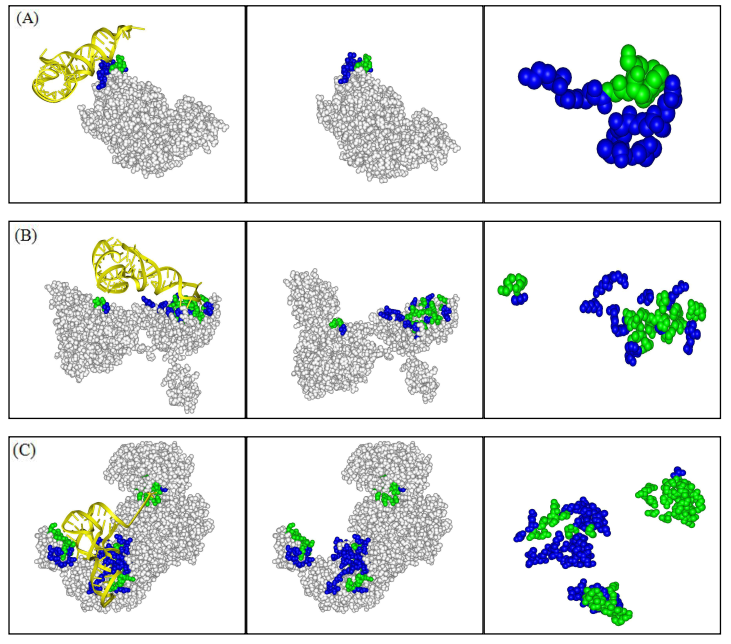


Figure S3. Multiple clusters of evolutionary conserved residues in protein interfaces. (A) In the complex of prolyl-tRNA synthetase from thermus thermophilus complexed with tRNA (PDB code 1h4q, chain A with *ρ* = 1.17), the interface contains one well-clustered region of conserved residues. (B) In the complex of tRNA synthetase complexed with tRNA (PDB code 2du3, chain A with *ρ* = 1.19), the interface contains two regions of conserved residues. (C) Three conserved clusters in the interface of E. coli leucyl-tRNA synthetase with tRNA (PDB code 4arc, chain A with *ρ* = 1.19). Figures show the protein domains as CPK (green and blue for conserved and other residues), the RNA domains as Stick (yellow).





Figure S4. Distribution of sub-cluster size (the number of interface residues in the conserved cluster).





Figure S5. Plot of ΔΔG values vs. sequence entropies for the 139 interface residues involved in 41 protein-RNA complexes for which experimental alanine scanning mutagenesis data are available.





Figure S6. Percentage distribution of the *ρ* values for all protein-RNA interfaces.
